# Chlomito: a novel tool for precise elimination of organelle genome contamination from nuclear genome assembly

**DOI:** 10.3389/fpls.2024.1430443

**Published:** 2024-08-27

**Authors:** Wei Song, Chong Li, Yanming Lu, Dawei Shen, Yunxiao Jia, Yixin Huo, Weilan Piao, Hua Jin

**Affiliations:** ^1^ Laboratory of Genetics and Disorders, Key Laboratory of Molecular Medicine and Biotherapy, Aerospace Center Hospital, School of Life Science, Beijing Institute of Technology, Beijing, China; ^2^ Research Institute for Science and Technology, Beijing Institute of Technology, Beijing, China; ^3^ Advanced Technology Research Institute, Beijing Institute of Technology, Jinan, China; ^4^ Department of Pathology, Aerospace Center Hospital, Beijing, China

**Keywords:** mitochondrial genome, chloroplast genome, chromosome-level assembly, organelle identification, horizontal gene transfer

## Abstract

**Introduction:**

Accurate reference genomes are fundamental to understanding biological evolution, biodiversity, hereditary phenomena and diseases. However, many assembled nuclear chromosomes are often contaminated by organelle genomes, which will mislead bioinformatic analysis, and genomic and transcriptomic data interpretation.

**Methods:**

To address this issue, we developed a tool named Chlomito, aiming at precise identification and elimination of organelle genome contamination from nuclear genome assembly. Compared to conventional approaches, Chlomito utilized new metrics, alignment length coverage ratio (ALCR) and sequencing depth ratio (SDR), thereby effectively distinguishing true organelle genome sequences from those transferred into nuclear genomes via horizontal gene transfer (HGT).

**Results:**

The accuracy of Chlomito was tested using sequencing data from Plum, Mango and *Arabidopsis*. The results confirmed that Chlomito can accurately detect contigs originating from the organelle genomes, and the identified contigs covered most regions of the organelle reference genomes, demonstrating efficiency and precision of Chlomito. Considering user convenience, we further packaged this method into a Docker image, simplified the data processing workflow.

**Discussion:**

Overall, Chlomito provides an efficient, accurate and convenient method for identifying and removing contigs derived from organelle genomes in genomic assembly data, contributing to the improvement of genome assembly quality.

## 1 Introduction

With the widespread application of high-throughput sequencing technology, researchers can rapidly obtain genomes of various species (Rhoads and Au, 2015; Goodwin et al., 2016; Jain et al., 2018). For chromosome-level *de-novo* genome assembly, long reads from third-generation sequencing (TGS) or short reads from second/next-generation sequencing (SGS/NGS) are first assembled into contigs. Then, these contigs are anchored into chromosomes based on Hi-C sequencing, thereby compleling nuclear chromosome assembly (Du et al., 2022). However, during genome assembly, the issue of organelle genome contamination often arises, where the sequences from mitochondrial DNA (mtDNA) and chloroplast DNA (cpDNA) are mistakenly assembled into nuclear genome. This phenomenon occurs because organelle DNA is co-extracted with nuclear DNA during genomic DNA purification, producing a mixed sequencing data set. Since hundreds copies of organelle genomes exist within a single cell with extremely smaller sizes compared to the nuclear genomes (Pyke, 1999), they are overrepresented in the sequencing data and high-frequently mis-assembled into nuclear genomes. Furthermore, organelle genome sequences exhibit significant similarity to certain nuclear genome sequences, particularly those organelle genes had transferred into nuclear genomes through horizontal gene transfer (HGT) (Martin, 2003; Timmis et al., 2004; Wei et al., 2022; Wang et al., 2024). Thus, it is challenging to accurately distinguish contigs derived from organelle genomes among all assembled contigs, especially those containing sequences transferred from the organelle genomes through HGT. Accurate identification and elimination of organelle genome sequences are essential for minimizing the contamination issue and will enhance the quality of genome assembly.

To identify and remove organelle genome contamination from nuclear genome assembly data, current methods primarily employ two approaches: the experimental removal prior to sequencing and the bioinformatic identification after assembly. For experimental removal, the density gradient centrifugation technique can be utilized to deplete organelle DNA during nuclear genomic DNA extraction, hence the contamination is reduced in downstream sequencing data (Lutz et al., 2011; Sikorskaite et al., 2013; Sandhya et al., 2020). However, the density gradient centrifugation is not easy to carry out, and requires a large amount of material for DNA extraction but generates low DNA yield. Thus, most reported *de-novo* genome assembly did not include this step. Also, experimental separation is often incomplete. Alternatively, for bioinformatic identification, the most widely adopted approach is to align assembled contigs to organelle reference genomes, followed by filtering based on alignment lengths (Howe et al., 2021; Mishra et al., 2021; Rhie et al., 2021; Zhang et al., 2024) or sequence similarity (Shirasawa et al., 2021; Bae et al., 2023; Yu et al., 2024). Though effective in reducing contamination, these computational approaches have limitations. It ignored potential HGT of organelle sequences into the nuclear genome (Cecchin et al., 2019; Allio et al., 2020; Kenny et al., 2020; Martin et al., 2023). Due to such transfer, the fragments of organelle genomes were inserted in the nuclear genomes, therefore, traditional sequence similarity-based methods hardly distinguish organelle genomes from the nuclear HGT regions. Additionally, current methods often require pre-assembled reference organelle genomes, limiting their applicability in the species without well-established organelle references. Moreover, the implementation of current methods generally lacks support from integrated and user-friendly software, requires users to manually perform all steps. The data processing is still time-consuming and prone to errors, particularly when dealing with large numbers of data sets.

To address the issue identifying organelle genome sequences from genomic assembly accurately, we established a novel method, which employed two key filtering criteria: the alignment length coverage ratio (ALCR) and sequencing depth ratio (SDR). The ALCR refers to the proportion of a contig’s total length that is aligned with the organelle reference genome relative to the total length of the contig. This criterion can differentiate contigs that contain only small pieces of organelle DNA, which more likely arise from HGT, as these fragments usually constitute only a small portion of the contig. Therefore, a low ALCR may indicate that the contig belongs to nuclear genome rather than organelle genome (Zhu et al., 2021; Nath et al., 2022; Hao et al., 2023; Zhou et al., 2023b). Meanwhile, the SDR refers to the ratio of each coting’s sequencing depth to the average sequencing depth of the organelle genome. Given that organelle genomes exist in many copies within a cell, they typically exhibit higher sequencing depths than nuclear genomes (Sanita Lima et al., 2016; Wang et al., 2018; Li et al., 2021; Giorgashvili et al., 2022; Zhou et al., 2023a). Therefore, a contig with a high sequencing depth ratio, similar to the average of the organelle genome, is more likely to be a part of the organelle genome. By combining these two metrics, we can significantly improve the accuracy of identifying and removing organelle genome sequences from genome assembly data.

Furthermore, to facilitate usage by researchers with limited bioinformatics experience, we have implemented this new approach as easy-to-use software and packaged it as a Docker image, enabling easy distribution and execution across diverse computing platforms with a single command. We validated the accuracy and reliability of our tool using sequencing data from Plum (*Prunus salicina*) (Liu et al., 2020), Mango (*Mangifera indica*) (Wang et al., 2020) and *Arabidopsis* (*Arabidopsis thaliana*) (Wang et al., 2022). Our software can not only accurately identify organelle genome contigs from genome assembly, but also accurately distinguish native organelle sequences from those inserted into the nuclear genome via HGT. Our tool will offer an accurate and effective solution for eliminating organelle DNA fragments from genome assembly contigs, hold significant merit in improving chromosome assembly, and deepen our understanding of the complex interactions between organelle and nuclear genomes.

## 2 Materials and methods

### 2.1 Availability of data and materials

To validate the accuracy of the Chlomito software in detecting organelle genome sequences, we utilized sequencing data of Mango and Plum from the NCBI Bioproject database, with accession numbers PRJNA487154 and PRJNA574159, respectively. The raw sequencing data for the PacBio HiFi reads and Illumina short reads of *Arabidopsis* were obtained from the National Genomics Data Center, Beijing Institute of Genomics, Chinese Academy of Sciences/China National Center for Bioinformation (GSA: CRA004538). These datasets include high-quality second and third-generation sequencing data, which were utilized for organelle genome identification and chromosome-level genome assembly.

### 2.2 Installation and implementation of Chlomito

Chlomito is Python (v3.8.5)-based software provided in the form of a Docker image. The image is accessible at https://hub.docker.com/repository/docker/songweidocker/chlomito. All analyses were conducted on an Ubuntu Linux 18.04.3 server, equipped with two Intel Xeon processors (32 cores each, totaling 64 threads) and 512 GB of RAM. The user manual for Chlomito is available on GitHub (https://github.com/songwei-hxb/chlomito).

Chlomito can be installed using the Docker v19.03.5 command:

docker pull songweidocker/chlomito:v1

The command for running Chlomito organelle genome identification and removing is as follow:

docker run –rm -v/var/run/docker.sock:/var/run/docker.sock -v `pwd`:/data -w/data songweidocker/chlomito:v1 chlomito -species plant -raw_genome genome_contigs.fasta -NGS_1 ngs_1.fastq -NGS_2 ngs_2.fastq -output identify_result -mito_ALCR_cutoff 0.5 -mito_SDR_cutoff 0.1 -chlo_ALCR_cutoff 0.5 -chlo_SDR_cutoff 0.1 -threads 60

### 2.3 Contig-level genome assembly

Flye v2.9 (Kolmogorov et al., 2019) is genome assembler software designed for long-read sequencing data from third-generation platforms such as PacBio and Oxford Nanopore. It is capable of assembling raw error-prone long reads into contiguous genomic sequences known as contigs. The goal of Flye is to generate high-quality genome assembly, especially for large or complex genomes. In this study, we utilized Flye to assemble PacBio sequencing data of Mango and Plum into genome assembly contigs. Due to the high error rate of TGS data in PacBio CLR reads and Nanopore reads, the assembled contigs were then corrected using Racon v1.3.1 (Vaser et al., 2017) and Pilon v1.22 (Walker et al., 2014).

### 2.4 Construction of organelle genome database

The mitochondria and chloroplast organelle genomes were firstly assembled from SGS data using GetOrganelle v1.7.1 (Jin et al., 2020). GetOrganelle is a powerful genomics software tool specifically designed for efficient assembly of mitochondrial and chloroplast genomes. It is capable of simultaneously assembling organelle genomes from both mitochondria and chloroplast. Compared to other similar software tools, GetOrganelle demonstrates superior performance in terms of both accuracy and speed for organelle genome assembly. After that, we merged the mitochondrial and chloroplast genomes published in the NCBI organelle database with organelle genomes assembled using GetOrganelle, and created a comprehensive local organelle genome database. Since the local database integrated existing public data resources with high-precision assembly outcomes, it could offer more comprehensive and accurate reference for organelle genomes.

### 2.5 The annotation of chloroplast and mitochondrial genomes

The chloroplast and mitochondria genome sequences were annotated with GeSeq (Tillich et al., 2017) and OGDRAW (Lohse et al., 2013). GeSeq pipeline analysis was performed using the annotation packages ARAGORN (Laslett and Canback, 2004), blatN (Kent, 2002), Chloe (Zhong, 2020) and HMMER (Eddy, 2011). GeSeq is a user-friendly online service specifically designed for the annotation of mitochondrial and chloroplast genomes. This platform enables researchers to upload unannotated DNA sequences and utilizes its database of existing high-quality annotations to identify and label genes, coding sequences, and other significant genomic features.

### 2.6 Calculation of alignment length coverage ratio

Following the construction of local organelle genome database, all contigs assembled by the Flye v2.9 software were aligned against this database using Minimap2 v2.17 (Li, 2018). Subsequent to the alignment process, Alignment Length Coverage Ratio (ALCR) was calculated for each contig. The core filtering criterion ALCR is defined as the ratio of the aligned length sum from a contig to its total length, which can be formulated as:


$$\begin{array}{c} \text{ALCR}\left(contig\right)\\ =\;\sum_{i=1}^{n}\text{aligned}\_\text{length}{\left(contig,ref\right)}_{i}\;/\;\text{total}\_\text{length}\left(contig\right) \end{array}$$

In the formula, ALCR(contig) is the ALCR value for a given contig, Σaligned_length(contig, ref) is the sum of lengths of all aligned regions (1 to n) between the contig and the reference organelle genome, and total_length(contig) is the total length of the contig. A higher ALCR value indicates greater similarity between the contig and the reference organelle genome, and thus a higher chance that the contig is from the organelle genome. Unlike previously reported methods, the calculation of ALCR does not solely rely on the single longest alignment region. Instead, it aggregates the lengths of all contig regions that align with the organelle genome reference. This approach offers a more comprehensive reflection of the alignment coverage between the contig and the organelle genomes. Finally, by comparing the aggregated alignment length of each contig against its total length, the alignment length coverage ratio for each contig is computed.

### 2.7 Calculation of sequencing depth ratio

The sequencing depth ratio (SDR) refers to the ratio between the sequencing depth of each contig and the average sequencing depth of the organelle genome, which can be formulated as:


SDR(contig)=depth(contig) / avg_depth(organelle_genome)

In the formula, SDR(contig) is the SDR value for a given contig, depth(contig) is the average SGS depth of the contig, and avg_depth(organelle_genome) is the average SGS depth of the organelle genome assembled by GetOrganelle. The average sequencing depth of the organelle genome is determined by aligning the SGS reads to the organelle genome assembled by GetOrganelle v1.7.1 using Bowtie2 v2.4.2 (Langmead and Salzberg, 2012), which generates a SAM file. This SAM file is then processed by Samtools v1.6 (Li et al., 2009) to produce a sorted BAM file with depth information. Finally, Bedtools v2.30.0 (Quinlan and Hall, 2010) is utilized to analyze this depth data and calculate the average sequencing depth across the organelle genome. The method for calculating the sequencing depth of each contig is identical to that used for the organelle genome. Upon completion of these calculations, the sequencing depth for each contig is divided by the average sequencing depth of the organelle genome to obtain SDR for each contig.

### 2.8 Identification of organelle sequences

After calculating the ALCR and SDR values for each contig using the locally constructed organelle genome database and SGS data, contigs belonging to the organelle genome are identified from genome assembly contigs based on ALCR and SDR filtering thresholds inputted by the user. Here, we used thresholds ALCR>0.5 and SDR>0.1 at the first round of filtering. The filtering thresholds can be further optimized according to the ALCR and SDR visualization scatter plot generated after running Chlomito. By utilizing adjusted filtering thresholds, the genome assembly contigs can be filtered and selected again, resulting in more precise outcomes.

### 2.9 Chromosomal-level genome assembly

The genome sizes of various species were calculated using jellyfish v2.2.10 (Marcais and Kingsford, 2011) and GenomeScope v2.0 (Vurture et al., 2017) with SGS data and input into Flye for contig-level genome assembly with TGS data. After contig-level genome assembly, contig sequences were corrected with SGS reads using racon v1.3.1 and pilon v1.22, and redundancy was reduced using purge_dups v1.2.5 (Guan et al., 2020). Hi-C sequencing data were aligned to the deduplicated contigs using HiC-Pro v3.1.0 (Servant et al., 2015), and finally, Allhic v0.9.8 (Zhang et al., 2019) was used to cluster, order, and orient the contigs based on Hi-C alignment results, achieving the final chromosomal-level genome assembly. Gaps or missing regions may be present in genome assembly due to the limitations of sequencing technologies. To obtain more complete and accurate genome sequences, we applied two approaches - Abyss Sealer v2.0.2 (Jackman et al., 2017) and TGS-GapCloser v1.1.1 (Xu et al., 2020) - for closing gaps in our chromosome-level genome assembly. TGS-GapCloser v1.1.1 utilizes long reads from TGS platforms to fill gaps between contigs and extend contig ends based on overlaps between contigs and long reads. Abyss Sealer v2.0.2 is a computational tool that seals gaps in genome assembly by aligning Illumina short reads to contig ends and performing local assembly to generate consensus sequences for gap regions. The methods applied in this study for chromosomal-level genome assembly are based on the previous study (Song et al., 2024).

## 3 Results

### 3.1 The development of Chlomito software

**Figure 1:**
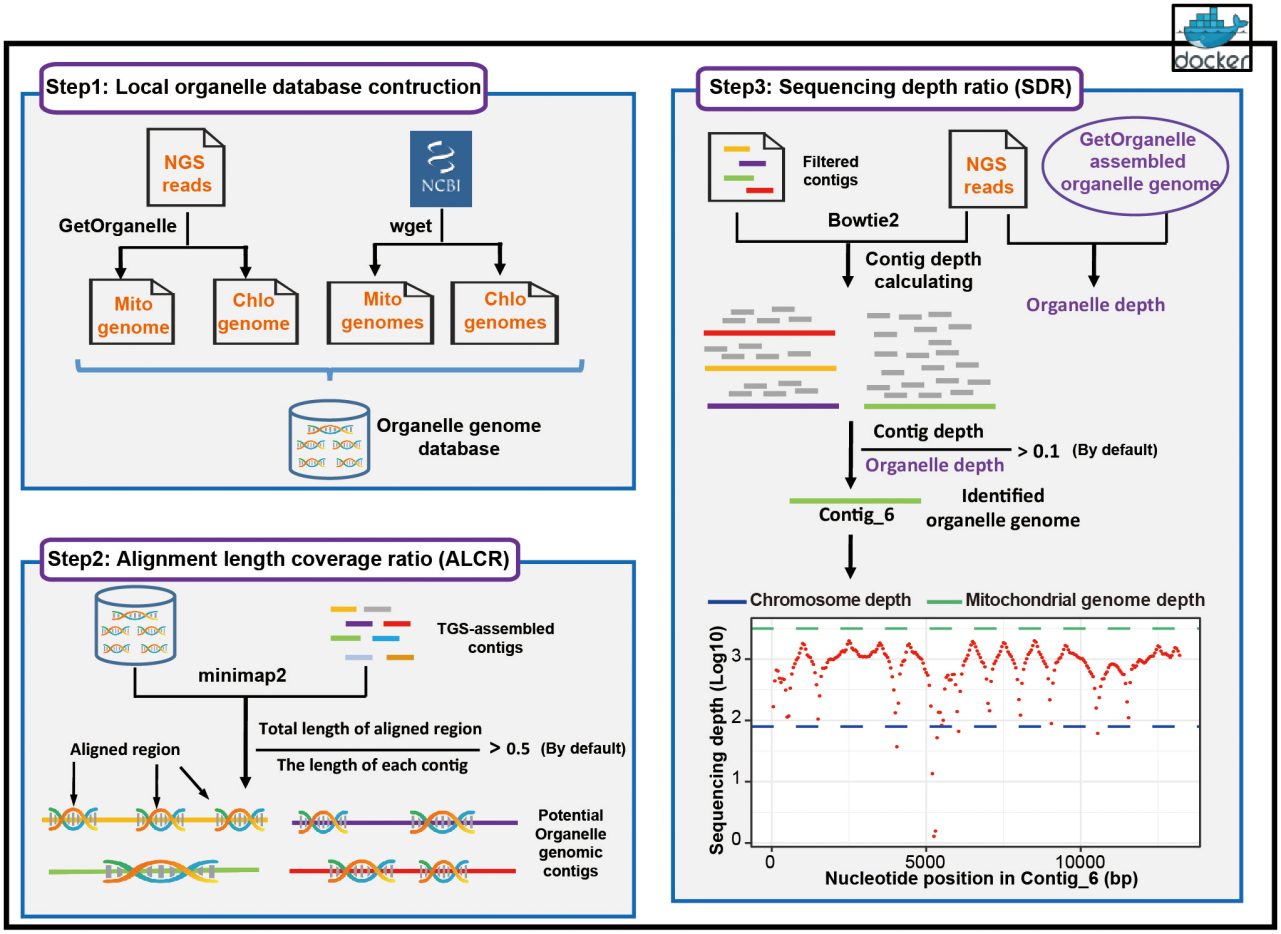
Workflow of the Chlomito software package.

Chlomito has two main functions, assembly of organelle genomes with NGS reads and screening contigs originated from organelle genomes. These functions are achieved through the workflow comprising three parts: the construction of local organelle genome database, organelle genome contig identification based on ALCR, and organelle genome contig identification based on SDR (**Figure 1**).

### 3.2 The construction of local organelle genome database

At the first step, Chlomito constructs a local organelle genome database by combining two approaches (**Figure 1**, step 1). The initial approach employs GetOrganelle to assemble mitochondrial and chloroplast genomes with SGS data. This is particularly valuable for the species without publicly-available organelle genome references, although the short-read assembly is sometimes incomplete. To further complement the database, the second approach downloads published high-quality organelle genomes from NCBI organelle database. With these approaches, the constructed local database is able to avoid the limitations from relying on a single data source, offering a broad and reliable organelle genome reference for downstream analysis.

### 3.3 Organelle genome identification based on ALCR

At the second step of Chlomito, organelle genome contigs are identified based on alignment length coverage ratio (ALCR). Chlomito preliminarily screens for potential organelle genomic sequences by aligning TGS-assembled genome contigs to the local database and filtering based on ALCR of the alignment (**Figure 1**, step 2). The core filtering criterion ALCR is defined as the ratio of the aligned length sum from a contig to its total length. Traditional methods typically consider only the longest length aligned between a contig and the organelle reference genome. In contrast, the alignment length of a contig in our approach is the sum of all aligned region lengths. This provides a more comprehensive assessment for the similarity between a contig and the organelle genome, improving the accuracy and sensitivity of organelle genome contig identification. In addition, compared to traditional methods, ALCR can also effectively distinguish organelle genome sequences from those inserted into the nuclear genome via HGT, as HGT insertions tend to be smaller, holding a lower ALCR value.

### 3.4 Organelle genome identification based on SDR

At the third step, Chlomito employs sequencing depth ratio (SDR) to further validate the organelle genome contigs previously filtered by the ALCR criteria (**Figure 1**, step 3). SDR refers to the ratio of the average sequencing depth of a contig to the average sequencing depth of the organelle genome. Given that the copy number of organelle genome is significantly higher in each cell compared to the nuclear genome, the sequencing depth ratio can be utilized to further distinguish organelle genomes from nuclear genomes. Considering the variable copy numbers of organelle genomes across various tissues and developmental stages (Preuten et al., 2010), it is difficult to accurately estimate the precise ratio of organelle to nuclear genome. Therefore, instead of using nuclear genome sequencing depth as a reference (Wang et al., 2018), the SDR approach adopts a method of comparing the sequencing depth of each contig against the average sequencing depth of organelle genomes to more accurately identify contigs derived from organelle genomes.

In summary, by utilizing both ALCR and SDR filtering methods, Chlomito can accurately identify organelle genome contigs from the total contigs. Furthermore, it can effectively reduce the misidentification of nuclear genome contigs as organelle genomes caused by HGT of organelle genomes.

### 3.5 The investigation into mitochondrial and chloroplast genomes in the NCBI database

**Figure 2:**
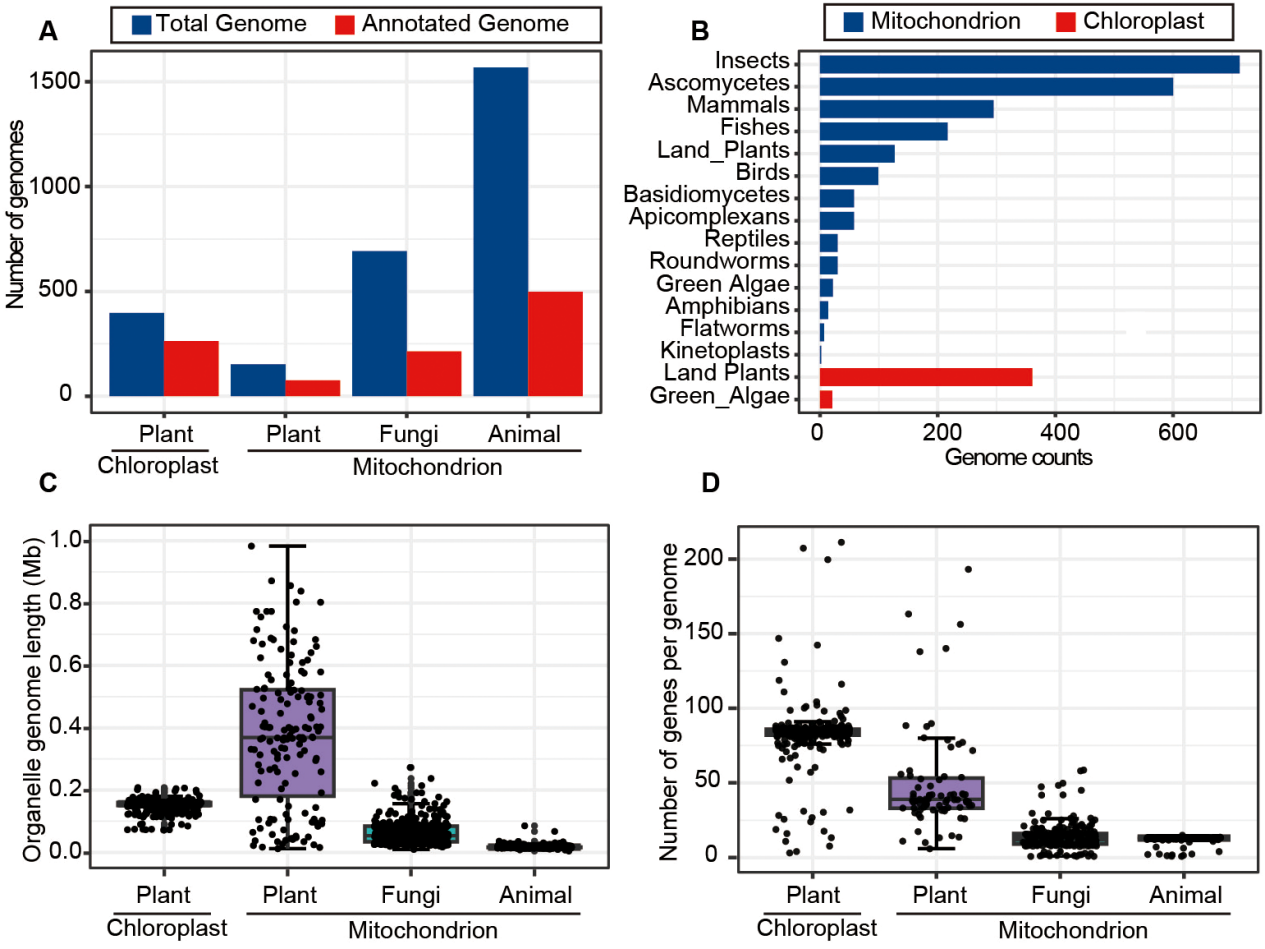
Overview of mitochondrial and chloroplast genome data in the NCBI organelle genome database. **(A)** Counts of the total and annotated mitochondrial and chloroplast genomes across typical kingdoms. **(B)** The numbers of mitochondrial and chloroplast genomes assembled for various taxonomic groups. **(C)** The length distribution of mitochondrial and chloroplast genomes for plants, animals and fungi. **(D)** The numbers of genes contained in mitochondrial and chloroplast genomes across different species.

To gain comprehensive understanding about the characteristics of mitochondrial and chloroplast genomes across a wide range of organisms, we explored genome sizes, gene numbers, and other features for mitochondrial and chloroplast genomes listed in the NCBI organelle database (https://www.ncbi.nlm.nih.gov/genome/browse#!/organelles/). The results revealed that the database contains 152 mitochondrial genomes (mitogenomes) and 263 chloroplast genomes (chlorogenomes) derived from plants, with approximately 50% to 60% of these genomes being annotated. In comparison, the numbers of animal and fungal mitogenomes were significantly higher, with 1,568 and 692 genomes respectively. However, the annotation rates for animal and fungal mitogenomes were lower, standing at only 30% (**Figure 2A**). Further inspection showed that most mitogenomes in the database were from insects in animal and ascomycetes in fungi, while chlorogenomes were predominantly from land plants and green algae (**Figure 2B**).

Plant chlorogenomes exhibited relative stability in terms of genome lengths and gene numbers, averaging around 0.15 Mb in size and containing ~82 genes on average (**Figures 2C, D**). In contrast, plant mitogenomes displayed greater variability in both lengths and gene numbers as previously reported (Bendich, 2010; Oldenburg and Bendich, 2015), suggesting the potential involvement of more complex evolutionary processes (Kubo and Newton, 2008). In terms of animal mitogenomes, we found a high degree of conservation, with an average size of 0.017 Mb and typically including 13 genes. Fungal mitogenomes, on the other hand, had an average size of 0.063 Mb and contained an average of 14 genes (**Figures 2C, D**). These analyses thoroughly characterized the features of organelle genome sizes and annotated status, as well as gene numbers, across different kingdoms such as Plantae, Animalia, and fungi, providing crucial support for effectively identifying and removing organelle genome segments from genomic assembly sequences in future research.

### 3.6 The performance of Chlomito on the detection of chloroplast genomes

**Figure 3:**
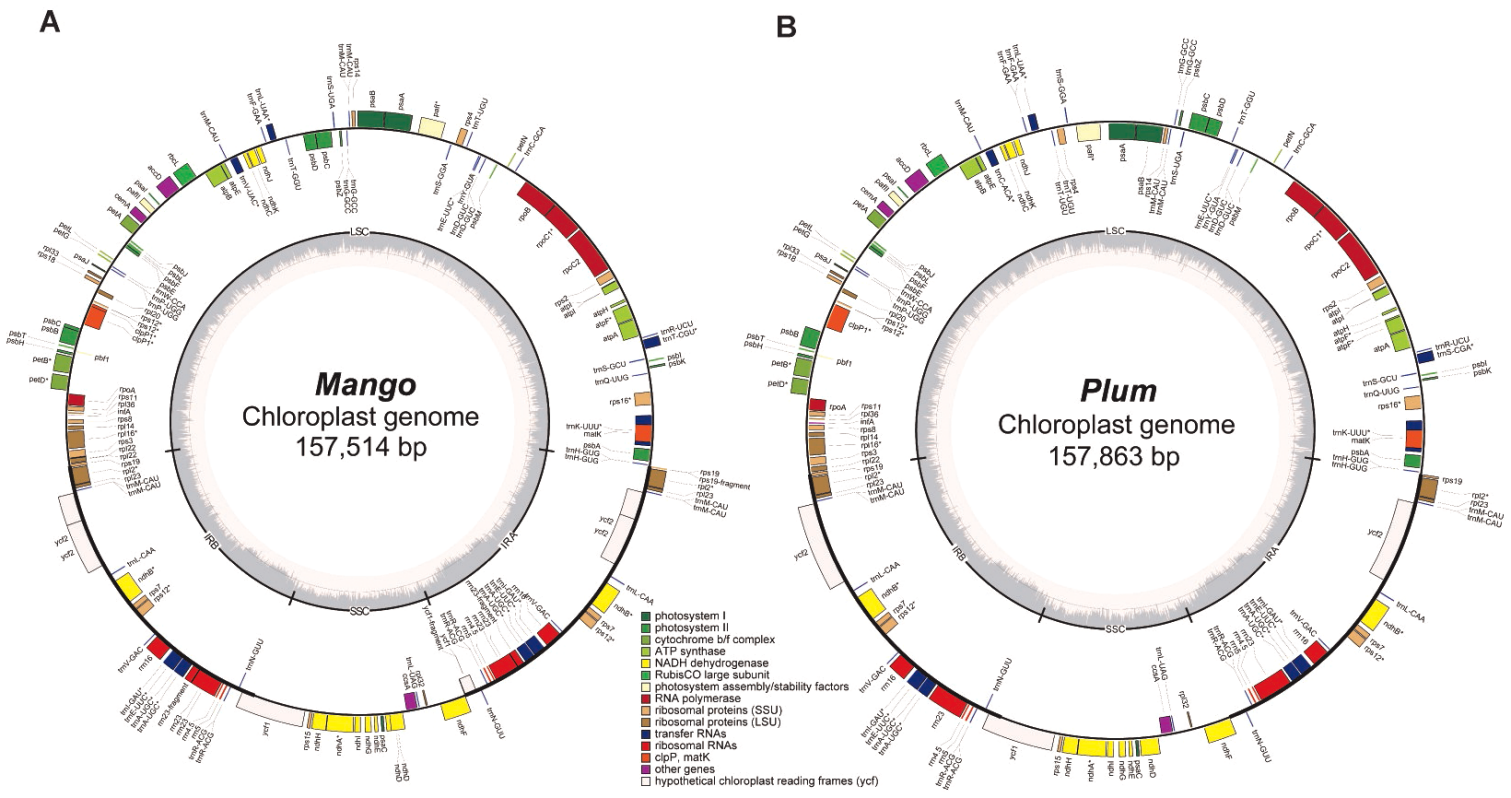
Chloroplast genome annotation of Mango **(A)** and Plum **(B)**.

To evaluate the performance of Chlomito in identifying chlorogenomes, we tested it with sequencing data derived from Plum (*Prunus salicina*) and Mango (*Mangifera indica*). Prior to detecting chloroplast genomic sequences from the contigs assembled from TGS reads, we first assembled the chlorogenomes of Mango and Plum from their NGS reads using Getorganelle respectively, and the assembly of each species generated a single sequence of complete chlorogenome. Collinearity analysis revealed high consistency between the assembled chlorogenomes and the published reference genomes in both Mango and Plum (**Supplementary Figure S1**). This demonstrated the accuracy and reliability of the Getorganelle assembly for downstream analysis. We then annotated the chlorogenomes of Mango and Plum using Geseq and found that they contained similar numbers of genes with highly similar arrangements (**Figure 3**). To investigate the structural conservation of chlorogenomes across diverse plant species, we compared the chlorogenomes of Mango and Plum with those of other plant species including *Arabidopsis thaliana* and *Zea mays*. The results showed that chlorogenomes were highly conserved in gene contents and orders across diverse plant species analyzed here (**Supplementary Figure S2**), implying that chlorogenomes may be structurally conserved across diverse plants.

**Figure 4:**
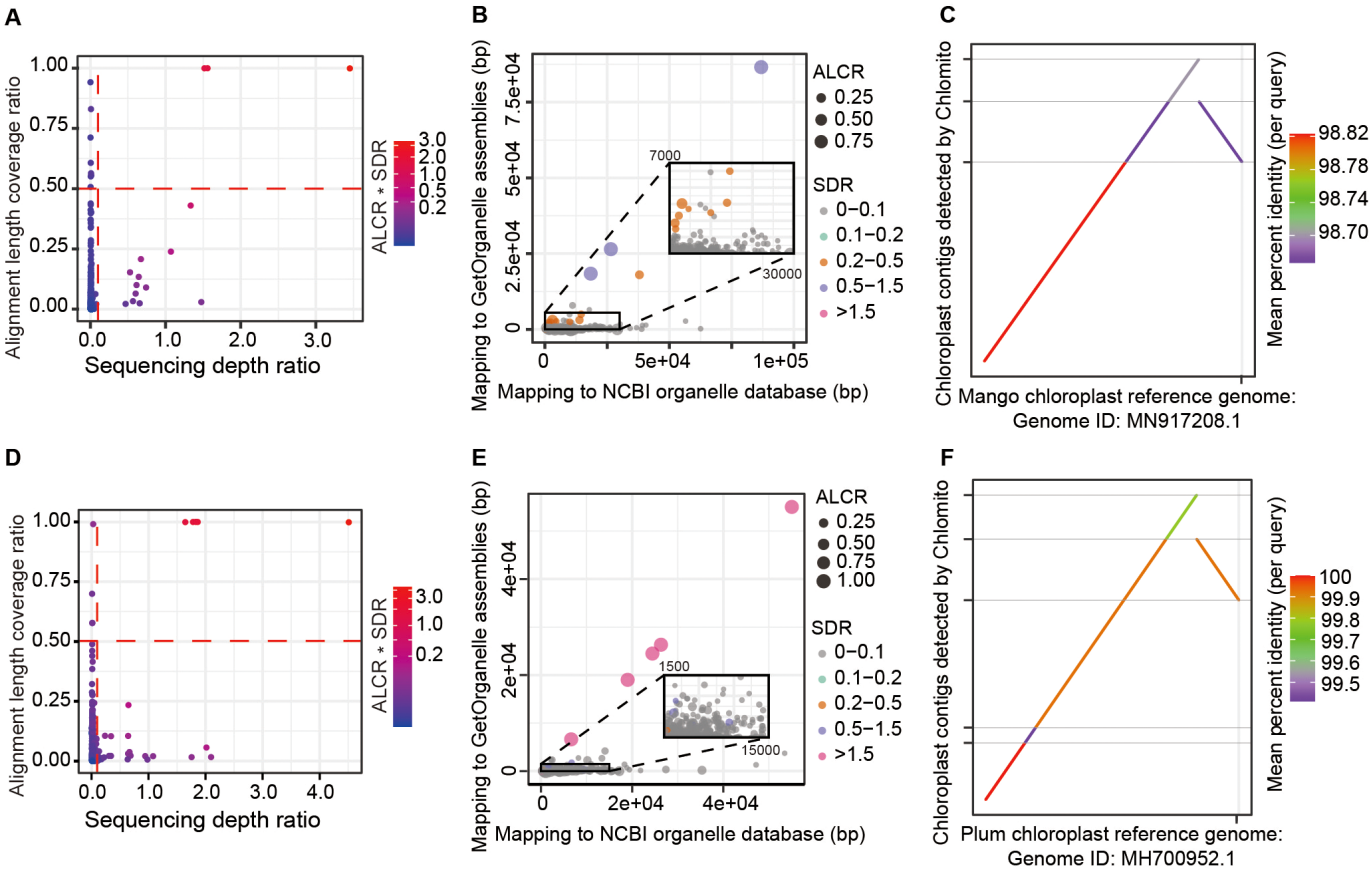
Chlomito accurately identifies chloroplast-derived contigs and validates their collinearity with chloroplast reference genomes from Mango **(A–C)** and Plum **(D–F)**. **(A, D)** Identification of chloroplast-derived contigs in Mango **(A)** and Plum **(D)** based on ALCR and SDR metrics. **(B, E)** Alignment lengths of Mango **(B)** and Plum **(E)** contigs with chloroplast genomes assembled using GetOrganelle and downloaded from NCBI database. **(C, F)** Collinearity analysis of Mango **(C)** and Plum **(F)** contigs identified by Chlomito against published chloroplast reference genomes.

After assembling the chlorogenomes of Mango and Plum using GetOrganelle, we integrated these sequences with the NCBI chlorogenome database to create a localized chloroplast database. Then, the TGS-assembled genome contigs were aligned to the local organelle genome database. We next employed two key metrics to identify chloroplast-derived contigs among the total TGS-assembled contigs. The first metric is Alignment Length Coverage Ratio (ALCR), which calculates the ratio of the aligned sum length of each contig to the total length of that contig. The second metric is Sequencing Depth Ratio (SDR), which computes the sequencing depth of each contig to the average sequencing depth of the assembled chlorogenome. Based on default parameters ALCR>0.5 and SDR>0.1, we identified 3 and 5 potential chloroplast-derived contigs from Mango and Plum samples respectively (**Figures 4A, D**). These contigs showed similar alignment lengths in the GetOrganelle-assembled and the NCBI database chlorogenomes (**Figures 4B, E**), further validating the reliability of the chloroplast genomic contigs detected by Chlomito. Collinearity analysis displayed excellent consistency between these identified contigs and the chloroplast reference genomes, and two inverted repeat regions of the chlorogenomes (IRA and IRB) were also clearly observed in the co-linearity analysis (**Figures 4C, F**). These results further confirmed that these contigs were indeed derived from the chlorogenomes and were completely detected.

In addition to the chloroplast-derived contigs, we also observed some contigs with low ALCR (<0.5) and high SDR (>0.1) in both Mango (11 contigs) and Plum (12 contigs) genomes. In Mango, 8 out of the 11 contigs with low ALCR and high SDR were confirmed to be of mitochondrial origin. These mitogenome contigs were detected during our chlorogenome contamination analysis, likely due to the occurrence of HGT between mitochondrial and chlorogenomes. Such gene transfer events can result in contigs with low ALCR and high SDR. In Plum, 5 of the 12 contigs were similarly identified as mitochondrial derivation. The remaining 7 contigs in Plum were subjected to further analysis using RepeatMasker, which revealed that the majority of these contigs contained repetitive sequences that constituted more than 50% of their lengths (**Supplementary Table S1**). This high proportion of repetitive sequences likely contributed to the unusually high sequencing depths observed in these contigs.

Based on the alignment results of all contigs from Mango and Plum against the local chloroplast genome database, we identified 226 Mango contigs and 174 Plum contigs that aligned with the database sequences at lengths greater than 5000 bp. However, the ratio of each contig’s length that aligned to the database (ALCR) was low, most of them had less than 10% coverage (**Supplementary Figure S3**). The chloroplast genomic fragments present in these ultra-long contigs may have been inserted into the nuclear chromosomes through HGT from the chloroplast genome. These results indicated that the traditional method of filtering out chlorogenomes based solely on alignment lengths might erroneously identify some ultra-long nuclear contigs that contain only a small proportion of chloroplast genomic content. Therefore, adopting more refined filtering criteria, such as ALCR and SDR, is critical to accurately differentiate contigs that are truly from the organelle genomes versus those inserted into nuclear genomes through HGT.

### 3.7 The performance of Chlomito on the detection of mitogenomes

Following the validation of Chlomito’s efficacy in chlorogenome identification, we expanded our investigation to assess its performance in detecting mitogenomes. Mitogenomes of Mango and Plum were first assembled from NGS data using GetOrganelle. Unlike a single sequence of complete chlorogenome assembled by Chlomito above (**Supplementary Figure S1**), the constructed mitogenomes of Mango and Plum were composed of multiple fragments. The assembled Mango mitogenome consisted of 15 sequences totaling 0.48 Mb, while Plum mitogenome was composed of 11 sequences totaling 0.36 Mb (**Supplementary Figure S4**). To validate the accuracy of the mitogenome assembly, we performed collinearity analysis between the assembled and the NCBI reference mitogenomes for Mango (MZ751075.1) and Plum (OK563724.1). The results showed that the assembled mitogenomes had high collinearity with the reference and covered most regions of the reference genomes (**Supplementary Figure S4**), indicating the high accuracy and completeness of the GetOrganelle-assembled mitogenomes. To evaluate the structural conservation of mitogenomes across different plant species, we annotated and compared the mitogenomes of Mango, Plum, *Zea mays*, and *Arabidopsis thaliana* with the OGDRAW website. The results showed that unlike chlorogenomes, the mitogenomes from these species did not show conserved gene contents or gene orders (**Supplementary Figure S5**). Subsequently, we integrated the assembled mitogenomes with those downloaded from NCBI database to establish a local database.

**Figure 5:**
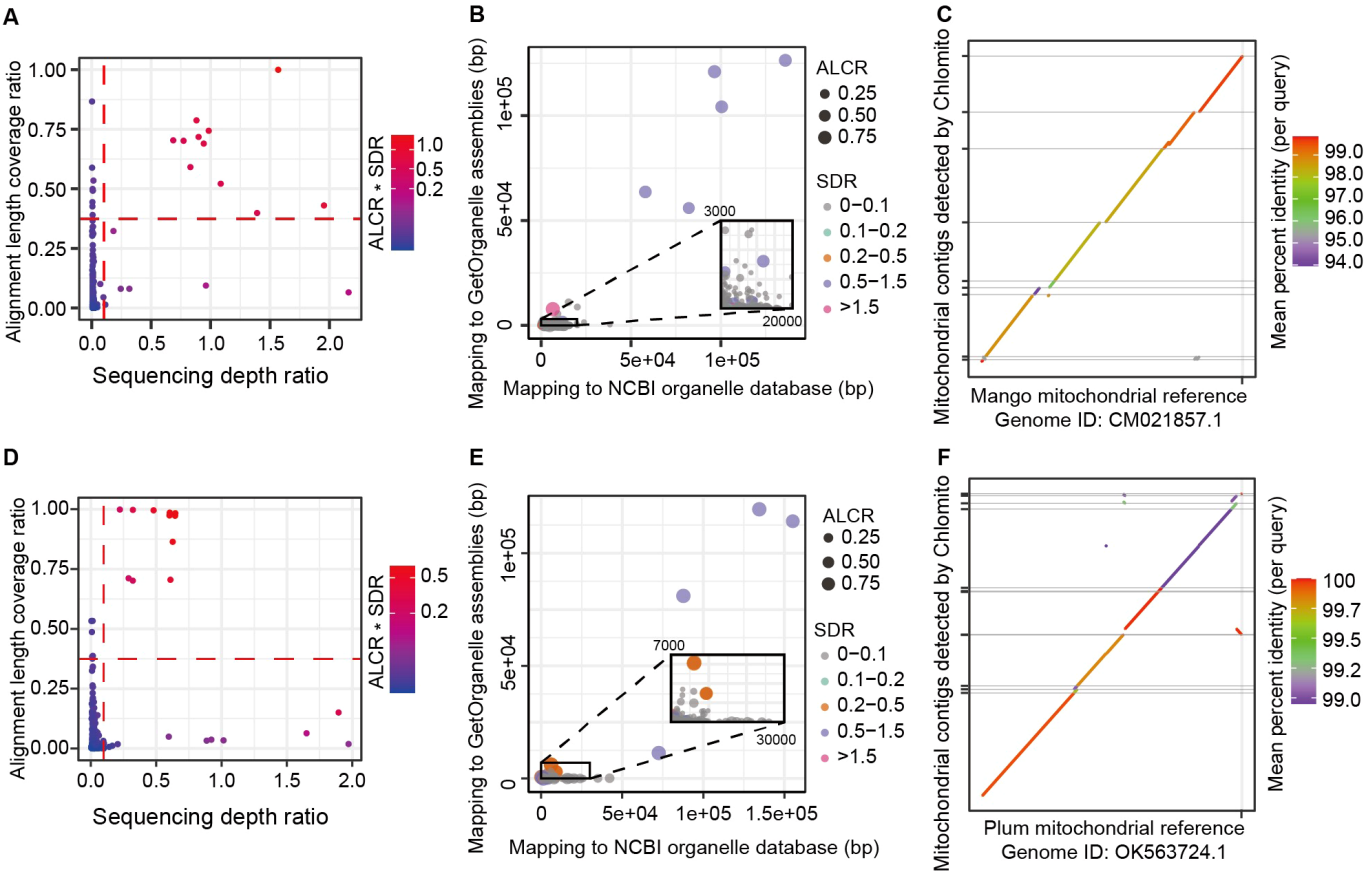
Chlomito accurately identifies mitochondrial-derived contigs and validates their collinearity with mitochondrial reference genomes from Mango **(A–C)** and Plum **(D–F)**. **(A, D)** Identification of mitochondria-derived contigs in Mango **(A)** and Plum **(D)** using ALCR and SDR metrics. **(B, E)** Alignment lengths of contigs with GetOrganelle-assembled and NCBI-downloaded mitogenomes for Mango **(B)** and Plum **(E)**. **(C, F)** Collinearity analysis of Chlomito-identified contigs against published mitochondrial reference genomes for Mango **(C)** and Plum **(F)**.

After constructing local mitogenome databases for Mango and Plum, we mapped SGS data to the mitogenomes in the local databases as well as to the TGS-assembled contigs, then calculated ALCR and SDR for each contig. Based on the parameters ALCR and SDR, we identified 11 and 10 contigs likely originating from the mitogenomes in Mango and Plum respectively (**Figures 5A, D**). These contigs also exhibited similar alignment lengths with the mitogenomes assembled by GetOrganelle and the NCBI database (**Figures 5B, E**). Moreover, collinearity analysis revealed that the identified contigs and the mitochondrial reference genomes had a high consistency in Mango and Plum, with the majority of the mitogenome regions being covered by these contigs (**Figures 5C, F**). Similar to the results in chloroplast, we also detected 116 and 52 contigs with alignment lengths exceeding 5000 bp but exhibiting low coverage (less than 10%) in Plum and Mango (**Supplementary Figure S6**). These results indicated that HGT of large fragments to the nucleus happened in both mitochondrial and chloroplast genomes.

### 3.8 The performance of Chlomito on the detection of HGT in *Arabidopsis*

To assess the accuracy of Chlomito in detecting HGT events, we utilized a experimentally-confirmed large nuclear insertion of mitochondrial DNA (numt) in *Arabidopsis* (Fields et al., 2022) as a test case, representing a HGT event from mitochondria to nuclei. This numt, located on chromosome 2 of *Arabidopsis thaliana*, spans approximately 641 kb and is one of the largest numts reported in plants to date. Its existence has been validated by fiber-based fluorescent *in situ* hybridization.

**Figure 6:**
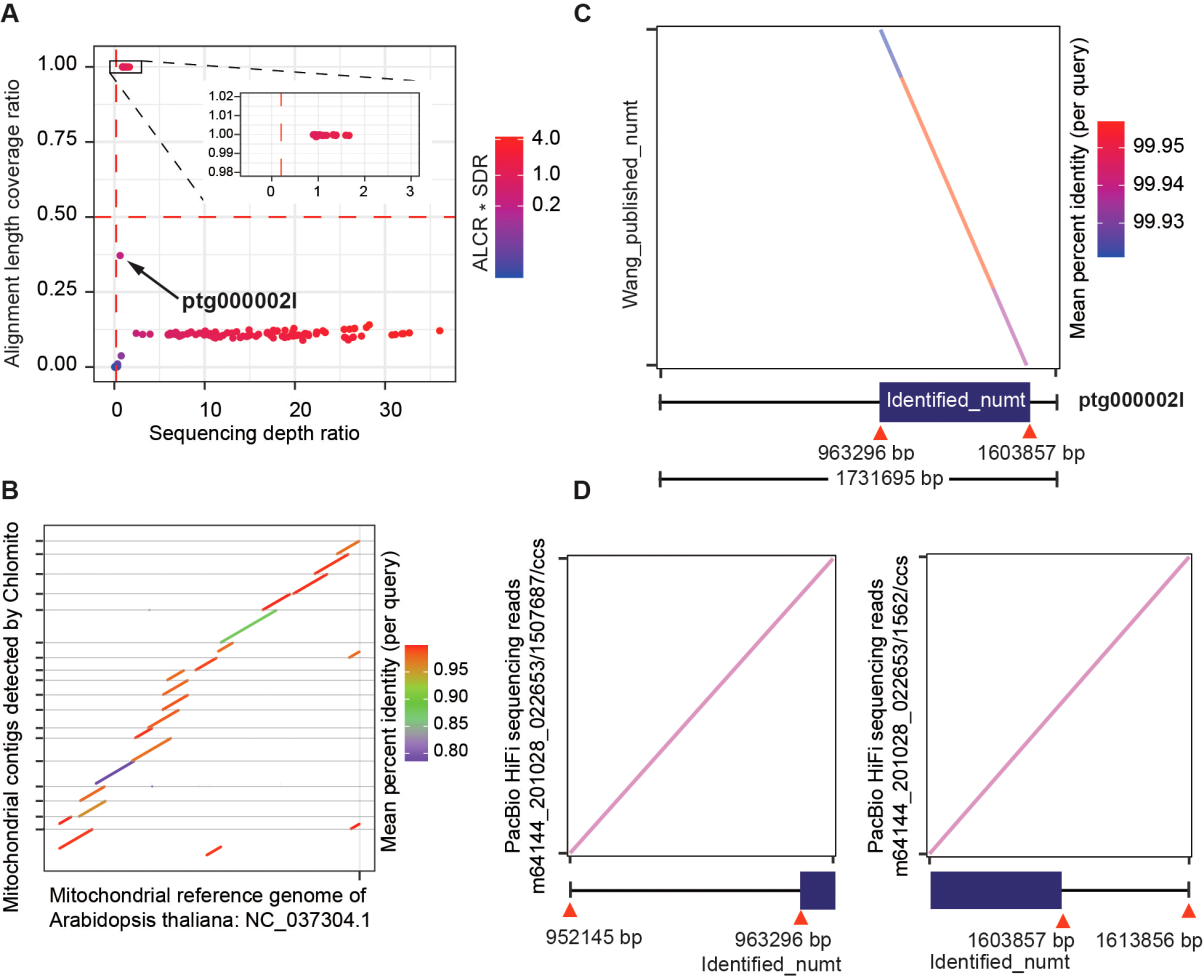
Chlomito accurately identifies mitochondria-derived contigs and a horizontally transferred organelle fragment (numt) from *Arabidopsis*. **(A)** Identification of mitochondria-derived contigs in *Arabidopsis* using ALCR and SDR as screening criteria. **(B)** Collinearity analysis of Chlomito-identified contigs against published mitochondrial reference genomes for *Arabidopsis*. **(C)** Comparison of the ptg000002l fragment with the previously reported large numt on chromosome 2 of *Arabidopsis*. **(D)** Alignment of PacBio HiFi sequences (Wang et al., 2022) with the numt and its flanking regions.

By applying ALCR and SDR as screening criteria, we identified a total of 22 TGS-assembled contigs that potentially belong to the *Arabidopsis* mitogenome (**Figure 6A**). Collinearity analysis of these fragments with the NCBI mitogenome reference revealed that these fragments exhibited high collinearity with the reference and covered most of the mitogenome regions (**Figure 6B**). These results again confirmed that Chlomito could effectively find out mitogenome contigs in *Arabidopsis*. Interestingly, we noticed one contig ptg0002l, which didn’t pass the mtDNA screening criteria of ALCR and SDR, meaning that it is not a mitogenome contig (**Figure 6A**). The alignment of ptg00021 with the local database showed a pretty high coverage rate but a low depth ratio, suggesting that it might be an HGT fragment. Surprisingly, comparison of ptg00021 with the previously validated large numt on chromosome 2 of *Arabidopsis* (Fields et al., 2022) revealed a perfect match between them (**Figure 6C**). Furthermore, we specifically aligned PacBio HiFi reads (Wang et al., 2022) to the junctions between the identified numt and its flanking regions on ptg0002l. We found that some HiFi reads span these junctions, which further substantiates that this numt was horizontally transferred into chromosome 2, rather than being the result of assembly errors (**Figure 6D**).

Similar to its performance in mitochondrial genome sequence detection, Chlomito accurately distinguished *Arabidopsis* chloroplast fragments from genomic contigs, as shown in **Supplementary Figure S7A**. The chloroplast genome fragments detected by Chlomito had similar alignment lengths with the reference genome assembled by GetOrganelle and the NCBI chloroplast reference genome (**Supplementary Figure S7B**). Furthermore, these detected chloroplast genome fragments showed good collinearity with the chloroplast reference genome and covered most of its regions (**Supplementary Figure S7C**). This further demonstrates Chlomito’s utility in accurately identifying organelle genome contigs within complex genomic datasets.

In summary, the Chlomito tool accurately identified the contigs of *Arabidopsis* mitogenome and chlorogenome, and also effectively distinguished genuine mitogenome fragments from numts, the HGT regions existed in nuclear genome. These results validated our method as a reliable tool for understanding complex genomic evolution.

### 3.9 Organelle genome contamination in chromosome assembly

**Figure 7:**
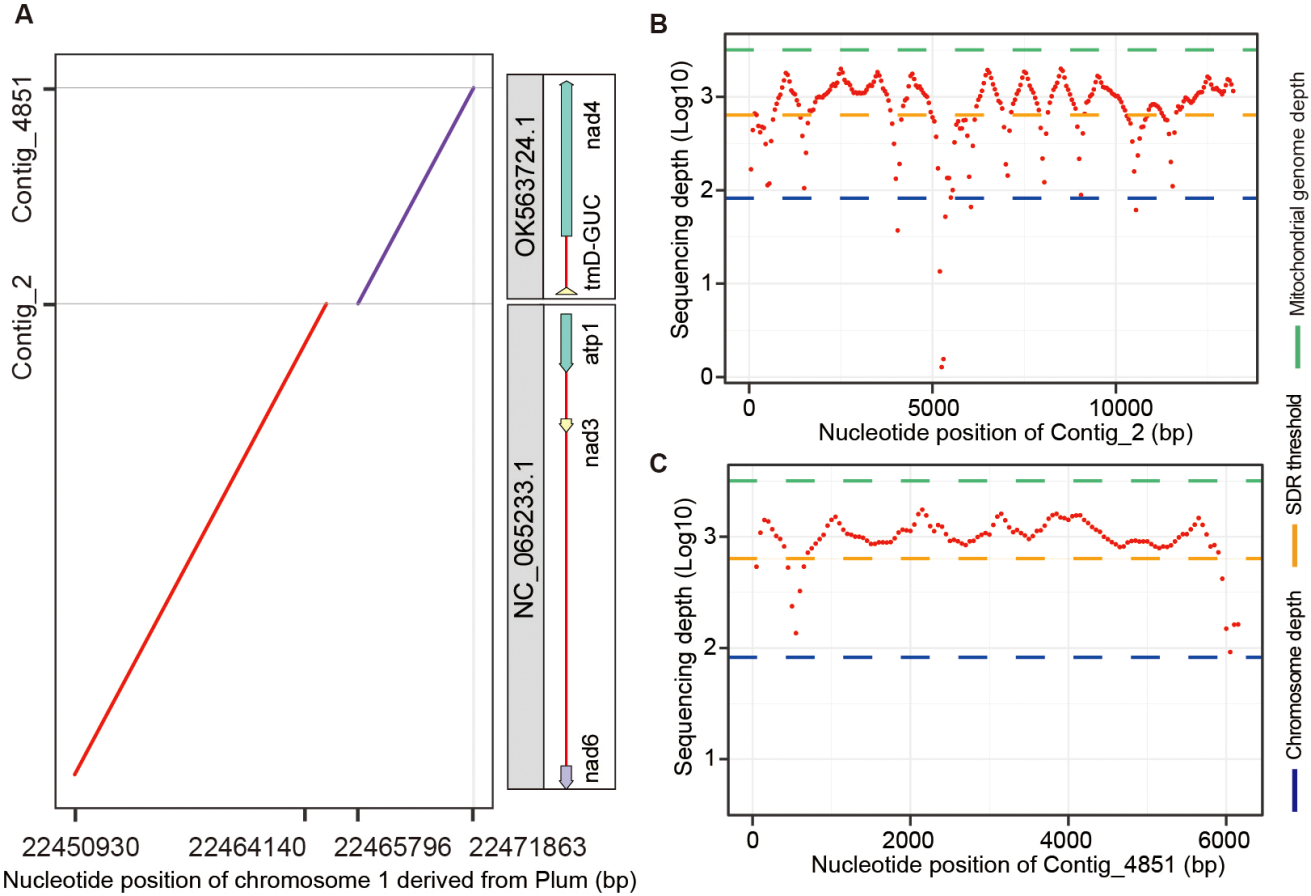
Identification and verification of organelle genome contamination in the assembled chromosome 1 of Plum. **(A)** The alignment between two mitochondria-derived contigs (identified by Chlomito) and the assembled chromosome 1 of Plum. **(B, C)** The sequencing depths are shown for contig_2 **(B)** and contig_4851 **(C)**, in parallel with the average depths of chromosome and mitochondrial genomes.

To evaluate the impact of organelle genome contamination on the accuracy of chromosome assembly, we aligned the Plum chromosomes assembled from all contigs without removing organelle sequences to the contigs identified as organelle DNA by Chlomito. The aligned result indicated that two mitogenome fragments (contig_2 and contig_4851) identified by Chlomito were erroneously assembled into chromosome 1 of Plum (**Figure 7A**). The full fragments of contig_2 (13,217 bp) and contig_4851 (6,187 bp) showed perfect matches with mitochondrial reference genomes NC_065233.1 and OK563724.1, which are complete mitogenomes of Plum listed in the NCBI organelle genome database. Further analysis demonstrated that the sequencing depths of these two contigs were much higher than the average depth of the chromosomal genome, and were close to the average depth of the mitogenome (**Figures 7B, C**). This is consistent with the characteristic that organelle genomes have higher copy numbers than nuclear genomes. In addition, we aligned PacBio sequencing reads to these two contigs and their flanking regions, and found that no PacBio reads could be mapped to their junction and flanking regions, further confirming that these two contigs were the result of chromosome assembly errors rather than true nuclear insertion of mitochondrial DNA (numt).

Altogether, these results highlight that unfiltered organelle sequences can truely contaminate the nuclear genome during chromosome-level genome assembly. Therefore, the prior identification and exclusion of organelle genome sequences using Chlomito are curcial for ensuring the accuracy and integrity of chromosome assembly.

## 4 Discussion

In this study, we have developed a novel tool called Chlomito that provides an innovative approach for accurately identifying organelle genome sequences from complex genomic assembly. This method significantly improves the accuracy of recognizing organelle genomic fragments by integrally applying two metrics. (1) The first metric is Alignment Length Coverage Ratio (ALCR). Different from previous conventional methods (Nath et al., 2022; Bao et al., 2024), the calculation of ALCR does not solely rely on the single longest alignment region. Instead, it adds the lengths of all regions on the contig that is aligned with the organelle genome database. This metric offers a more accurate and comprehensive reflection of the alignment coverage between the contig and the organelle genome. The introduction of ALCR can significantly reduce the likelihood of incorrectly identifying nuclear genomic sequences as organelle genome sequences, especially for those organelle genome sequences that have been inserted into nuclear genomes via horizontal gene transfer (HGT). (2) The second metric is Sequencing Depth Ratio (SDR). Considering the varying copy numbers of organelle genomes in different tissues and developmental stages of plants (Preuten et al., 2010), the ratio of sequencing depths between organelle genome and nuclear genome is not constant (Wang et al., 2018). Consequently, in this study, we compared the sequencing depths of contigs against the average sequencing depth of organelle genome to enhance the precision of detection outcomes. The application of SDR provides an additional robust filtering dimension, further ensures the identification of sequences truly belonging to organelle genomes among all the assembled contigs.

Moreover, we have noted that recently, some new methods such as ODNA (Martin et al., 2023) and Odintifier (Samaniego Castruita et al., 2015) can also be used for the identification of organelle genomes. ODNA takes a machine learning approach, extracting features from the sequences and training classification models to distinguish organelle and nuclear genome sequences. Odintifier utilizes phasing technology to separate reads containing SNVs into organelle and nuclear genome reads, thereby enabling the identification of organelle genome sequences. Compared to them, Chlomito not only considers sequence alignment information but also takes full advantage of the differences in copy numbers and sequencing depths between organelle and nuclear genomes, thereby enhancing the identification accuracy. The organelle genome contigs identified by Chlomito exhibit high collinearity with its reference and cover most of the reference genome regions (**Figures 4C, F**, **5C, F**, **6B**; **Supplementary Figure S7**), as we tested it using sequencing data from multiple species, including Mango (*Mangifera indica*) (Wang et al., 2020), Plum (*Prunus salicina*) (Liu et al., 2020), and *Arabidopsis* (*Arabidopsis thaliana*) (Wang et al., 2022). Thus, Chlomito is capable of accurately detecting the organelle genome contaminants scattered in the assembly results. Also, it can effectively pick out the entire organelle genome sequences (**Figures 4C, F**). In short, Chlomito is a reliable tool in organelle genomic studies, and in supporting precise nuclear genome assembly by removing organelle genome contaminants.

In addition to the contigs identified by Chlomito as belonging to organelle genomes, there were also numerous contigs with alignment lengths to the reference organelle genomes exceeding 5 kb but exhibiting low alignment coverage (ALCR less than 10%) relative to the contig lengths (**Supplementary Figure S6**). This phenomenon may be attributable to the intracellular insertion of organelle genomes into the nuclear genome via HGT events. HGT is a significant mechanism in the evolutionary process, particularly in the exchange of genetic information between organelle genomes and host nuclear genomes. Similar studies have also observed the phenomenon of large organelle genome fragments over 4 kb being transferred into nuclear genomes via HGT in watermelon and melon (Cui et al., 2021). These findings suggest that large-scale HGT may be a widespread occurrence across diverse species. In this context, it is particularly important to accurately distinguish sequence exchanges between organelle and nuclear genomes caused by HGT. Chlomito is designed to address this challenge by employing two powerful metrics — ALCR and SDR. Two-dimensional grouping and filtering in Chlomito with ALCR and SDR can clearly separate different groups: the organellel genome contigs and the nuclear insertions of organellel sequences (**Figures 4A, D**, **5A, D**, **6A**). Additionally, some contigs with low ALCR (<0.5) and high SDR (>0.5) may be the result of either horizontal gene transfer between mitochondrial and chloroplast genomes or a high proportion of repetitive sequences within the contigs. To improve the accuracy of detection, users can initially run Chlomito with lower filtering thresholds and then determine more precise filtering thresholds using the generated ALCR and SDR visualization scatter plot for the following run. The application of Chlomito will deepen our understanding of the complex interactions among mitochondrial, chloroplast, and nuclear genomes through HGT.

The organelle genome contaminants greatly affect chromosome-level genome assembly results. When chromosomes were assembled using all contigs without removing organelle genomes, organelle genome segments were erroneously inserted into chromosomes by genome assembly software (**Figure 7**). Therefore, identifying and eliminating organelle genome contamination prior to chromosomal-level assembly are critical to ensure the fidelity of the assembly outcomes. The development of tools like Chlomito is important for improving the quality and reliability of chromosomal-level genome assembly in scientific research.

In summary, the development of Chlomito offers a precise and efficient approach for detecting and filtering organelle DNA sequences from genome assembly contigs, which significantly contributes to enhancing the quality of chromosome assembly. Furthermore, as Chlomito is capable of effectively distinguishing genuine organelle genome sequences from what have been integrated into the nuclear genome via HGT, it will facilitate broad investigation into the mechanisms of genetic exchange between chromosomal and organelle genomes across a wide range of species in the future, offering new insights on the dynamic changes and evolutionary processes of organelle genomes.

## Data Availability

To validate the accuracy of the Chlomito software in detecting organelle genome sequences, we utilized sequencing data of Mango and Plum from the NCBI Bioproject database, with accession numbers PRJNA487154 and PRJNA574159, respectively. The raw sequencing data for the PacBio HiFi reads and Illumina short reads of Arabidopsis were obtained from the National Genomics Data Center, Beijing Institute of Genomics, Chinese Academy of Sciences/China National Center for Bioinformation (GSA: CRA004538). These datasets include high-quality second and third-generation sequencing data, which were utilized for organelle genome identification and chromosome-level genome assembly.

## Supplementary material

Supplementary Figure 1 High collinearity between GetOrganelle-assembled chloroplast genomes and NCBI reference genomes. The results from Mango **(A)** and Plum **(B)** are shown.

Supplementary Figure 2 Annotation and comparison of chloroplast genomes of Mango, Plum, *Arabidopsis*, and *Zea mays*. The chloroplast genomes of these species exhibit high conservation in gene contents and order.

Supplementary Figure 3 Analysis of contigs with chloroplast reference genome alignments in Mango **(A)** and Plum **(B)** samples. The alignment length coverage ratio (ALCR, blue bars), sequencing depth ratio (SDR, orange bars), and contig alignment lengths with chloroplast reference genomes are shown for contigs with varying alignment lengths.

Supplementary Figure 4 Collinearity comparison of mitochondrial genomes of Mango **(A)** and Plum **(B)** assembled by GetOrganelle with mitochondrial reference genomes from NCBI.

Supplementary Figure 5 Annotation and comparison of mitochondrial genomes of Mango, Plum, *Arabidopsis*, and *Zea mays*. In contrast to the conserved chloroplast genomes (**Supplementary Figure S2**), the mitochondrial genomes of these species show significant variations in gene content and gene order.

Supplementary Figure 6 Analysis of contigs with mitochondrial reference genome alignments in Mango **(A)** and Plum **(B)** samples. The alignment length coverage ratio (ALCR, blue bars), sequencing depth ratio (SDR, orange bars), and contig alignment lengths with mitochondrial reference genomes are shown for contigs with varying alignment lengths.

Supplementary Figure 7 Chlomito accurately identifies chloroplast-derived contigs and validates their collinearity with chloroplast reference genome from *Arabidopsis*. **(A)** Identification of chloroplast-derived contigs in *Arabidopsis* assembly based on ALCR and SDR metrics. **(B)** Alignment lengths of *Arabidopsis* contigs with chloroplast genomes assembled using GetOrganelle and downloaded from NCBI database. **(C)** Collinearity analysis of *Arabidopsis* contigs identified by Chlomito against published *Arabidopsis* chloroplast reference genome.

Supplementary Table 1 Repetitive sequences identified using RepeatMasker in the Plum contigs with low ALCR and high SDR.

## References

[B1] Allio R. Schomaker-Bastos A. Romiguier J. Prosdocimi F. Nabholz B. Delsuc F. (2020). MitoFinder: Efficient automated large-scale extraction of mitogenomic data in target enrichment phylogenomics. Mol. Ecol. Resour. 20, 892–905. doi: 10.1111/1755-0998.13160 32243090 PMC7497042

[B2] Bae E. K. Kang M. J. Lee S. J. Park E. J. Kim K. T. (2023). Chromosome-level genome assembly of the Asian aspen Populus davidiana Dode. Sci. Data 10, 431. doi: 10.1038/s41597-023-02350-5 37414813 PMC10326025

[B3] Bao Y. Zhang Q. Huang J. Zhang S. Yao W. Yu Z.. (2024). A chromosomal-scale genome assembly of modern cultivated hybrid sugarcane provides insights into origination and evolution. Nat. Commun. 15, 3041. doi: 10.1038/s41467-024-47390-6 38589412 PMC11001919

[B4] Bendich A. J. (2010). Mitochondrial DNA, chloroplast DNA and the origins of development in eukaryotic organisms. Biol. Direct 5, 42. doi: 10.1186/1745-6150-5-42 20587059 PMC2907347

[B5] Cecchin M. Marcolungo L. Rossato M. Girolomoni L. Cosentino E. Cuine S.. (2019). Chlorella vulgaris genome assembly and annotation reveals the molecular basis for metabolic acclimation to high light conditions. Plant J. 100, 1289–1305. doi: 10.1111/tpj.14508 31437318 PMC6972661

[B6] Cui H. Ding Z. Zhu Q. Wu Y. Qiu B. Gao P. (2021). Comparative analysis of nuclear, chloroplast, and mitochondrial genomes of watermelon and melon provides evidence of gene transfer. Sci. Rep. 11, 1595. doi: 10.1038/s41598-020-80149-9 33452307 PMC7811005

[B7] Du Y. Song W. Yin Z. Wu S. Liu J. Wang N.. (2022). Genomic analysis based on chromosome-level genome assembly reveals an expansion of terpene biosynthesis of azadirachta indica. Front. Plant Sci. 13. doi: 10.3389/fpls.2022.853861 PMC9069239 35528946

[B8] Eddy S. R. (2011). Accelerated profile HMM searches. PloS Comput. Biol. 7, e1002195. doi: 10.1371/journal.pcbi.1002195 22039361 PMC3197634

[B9] Fields P. D. Waneka G. Naish M. Schatz M. C. Henderson I. R. Sloan D. B. (2022). Complete sequence of a 641-kb insertion of mitochondrial DNA in the arabidopsis thaliana nuclear genome. Genome Biol. Evol. 14 (5). doi: 10.1093/gbe/evac059 PMC9071559 35446419

[B10] Giorgashvili E. Reichel K. Caswara C. Kerimov V. Borsch T. Gruenstaeudl M. (2022). Software choice and sequencing coverage can impact plastid genome assembly-A case study in the narrow endemic calligonum bakuense. Front. Plant Sci. 13. doi: 10.3389/fpls.2022.779830 PMC9296850 35874012

[B11] Goodwin S. McPherson J. D. McCombie W. R. (2016). Coming of age: ten years of next-generation sequencing technologies. Nat. Rev. Genet. 17, 333–351. doi: 10.1038/nrg.2016.49 27184599 PMC10373632

[B12] Guan D. McCarthy S. A. Wood J. Howe K. Wang Y. Durbin R. (2020). Identifying and removing haplotypic duplication in primary genome assemblies. Bioinformatics 36, 2896–2898. doi: 10.1093/bioinformatics/btaa025 31971576 PMC7203741

[B13] Hao F. Liu X. Zhou B. Tian Z. Zhou L. Zong H.. (2023). Chromosome-level genomes of three key Allium crops and their trait evolution. Nat. Genet. 55, 1976–1986. doi: 10.1038/s41588-023-01546-0 37932434

[B14] Howe K. Chow W. Collins J. Pelan S. Pointon D. L. Sims Y.. (2021). Significantly improving the quality of genome assemblies through curation. Gigascience 10 (1), 1–9. doi: 10.1093/gigascience/giaa153 PMC7794651 33420778

[B15] Jackman S. D. Vandervalk B. P. Mohamadi H. Chu J. Yeo S. Hammond S. A.. (2017). ABySS 2.0: resource-efficient assembly of large genomes using a Bloom filter. Genome Res. 27, 768–777. doi: 10.1101/gr.214346.116 28232478 PMC5411771

[B16] Jain M. Koren S. Miga K. H. Quick J. Rand A. C. Sasani T. A.. (2018). Nanopore sequencing and assembly of a human genome with ultra-long reads. Nat. Biotechnol. 36, 338–345. doi: 10.1038/nbt.4060 29431738 PMC5889714

[B17] Jin J. J. Yu W. B. Yang J. B. Song Y. dePamphilis C. W. Yi T. S.. (2020). GetOrganelle: a fast and versatile toolkit for accurate *de novo* assembly of organelle genomes. Genome Biol. 21, 241. doi: 10.1186/s13059-020-02154-5 32912315 PMC7488116

[B18] Kenny N. J. McCarthy S. A. Dudchenko O. James K. Betteridge E. Corton C.. (2020). The gene-rich genome of the scallop Pecten maximus. Gigascience 9 (5), 1–13. doi: 10.1093/gigascience/giaa037 PMC7191990 32352532

[B19] Kent W. J. (2002). BLAT–the BLAST-like alignment tool. Genome Res. 12, 656–664. doi: 10.1101/gr.229202 11932250 PMC187518

[B20] Kolmogorov M. Yuan J. Lin Y. Pevzner P. A. (2019). Assembly of long, error-prone reads using repeat graphs. Nat. Biotechnol. 37, 540–546. doi: 10.1038/s41587-019-0072-8 30936562

[B21] Kubo T. Newton K. J. (2008). Angiosperm mitochondrial genomes and mutations. Mitochondrion 8, 5–14. doi: 10.1016/j.mito.2007.10.006 18065297

[B22] Langmead B. Salzberg S. L. (2012). Fast gapped-read alignment with Bowtie 2. Nat. Methods 9, 357–359. doi: 10.1038/nmeth.1923 22388286 PMC3322381

[B23] Laslett D. Canback B. (2004). ARAGORN, a program to detect tRNA genes and tmRNA genes in nucleotide sequences. Nucleic Acids Res. 32, 11–16. doi: 10.1093/nar/gkh152 14704338 PMC373265

[B24] Li H. (2018). Minimap2: pairwise alignment for nucleotide sequences. Bioinformatics 34, 3094–3100. doi: 10.1093/bioinformatics/bty191 29750242 PMC6137996

[B25] Li H. Handsaker B. Wysoker A. Fennell T. Ruan J. Homer N.. (2009). The sequence alignment/map format and SAMtools. Bioinformatics 25, 2078–2079. doi: 10.1093/bioinformatics/btp352 19505943 PMC2723002

[B26] Li S. Chang L. Zhang J. (2021). Advancing organelle genome transformation and editing for crop improvement. Plant Commun. 2, 100141. doi: 10.1016/j.xplc.2021.100141 33898977 PMC8060728

[B27] Liu C. Feng C. Peng W. Hao J. Wang J. Pan J.. (2020). Chromosome-level draft genome of a diploid plum (Prunus salicina). Gigascience 9 (12), 1–11. doi: 10.1093/gigascience/giaa130 PMC7727024 33300949

[B28] Lohse M. Drechsel O. Kahlau S. Bock R. (2013). OrganellarGenomeDRAW–a suite of tools for generating physical maps of plastid and mitochondrial genomes and visualizing expression data sets. Nucleic Acids Res. 41, W575–W581. doi: 10.1093/nar/gkt289 23609545 PMC3692101

[B29] Lutz K. A. Wang W. Zdepski A. Michael T. P. (2011). Isolation and analysis of high quality nuclear DNA with reduced organellar DNA for plant genome sequencing and resequencing. BMC Biotechnol. 11, 54. doi: 10.1186/1472-6750-11-54 21599914 PMC3131251

[B30] Marcais G. Kingsford C. (2011). A fast, lock-free approach for efficient parallel counting of occurrences of k-mers. Bioinformatics 27, 764–770. doi: 10.1093/bioinformatics/btr011 21217122 PMC3051319

[B31] Martin R. Nguyen M. K. Lowack N. Heider D. (2023). ODNA: identification of organellar DNA by machine learning. Bioinformatics 39 (5), btad326. doi: 10.1093/bioinformatics/btad326 37195463 PMC10229373

[B32] Martin W. (2003). Gene transfer from organelles to the nucleus: frequent and in big chunks. Proc. Natl. Acad. Sci. U.S.A. 100, 8612–8614. doi: 10.1073/pnas.1633606100 12861078 PMC166356

[B33] Mishra B. Ulaszewski B. Meger J. Aury J. M. Bodenes C. Lesur-Kupin I.. (2021). A chromosome-level genome assembly of the european beech (Fagus sylvatica) reveals anomalies for organelle DNA integration, repeat content and distribution of SNPs. Front. Genet. 12. doi: 10.3389/fgene.2021.691058 PMC8862710 35211148

[B34] Nath O. Fletcher S. J. Hayward A. Shaw L. M. Masouleh A. K. Furtado A.. (2022). A haplotype resolved chromosomal level avocado genome allows analysis of novel avocado genes. Hortic. Res. 9, uhac157. doi: 10.1093/hr/uhac157 36204209 PMC9531333

[B35] Oldenburg D. J. Bendich A. J. (2015). DNA maintenance in plastids and mitochondria of plants. Front. Plant Sci. 6. doi: 10.3389/fpls.2015.00883 PMC4624840 26579143

[B36] Preuten T. Cincu E. Fuchs J. Zoschke R. Liere K. Borner T. (2010). Fewer genes than organelles: extremely low and variable gene copy numbers in mitochondria of somatic plant cells. Plant J. 64, 948–959. doi: 10.1111/j.1365-313X.2010.04389.x 21143676

[B37] Pyke K. A. (1999). Plastid division and development. Plant Cell 11, 549–556. doi: 10.1105/tpc.11.4.549 10213777 PMC144208

[B38] Quinlan A. R. Hall I. M. (2010). BEDTools: a flexible suite of utilities for comparing genomic features. Bioinformatics 26, 841–842. doi: 10.1093/bioinformatics/btq033 20110278 PMC2832824

[B39] Rhie A. McCarthy S. A. Fedrigo O. Damas J. Formenti G. Koren S.. (2021). Towards complete and error-free genome assemblies of all vertebrate species. Nature 592, 737–746. doi: 10.1038/s41586-021-03451-0 33911273 PMC8081667

[B40] Rhoads A. Au K. F. (2015). PacBio sequencing and its applications. Genomics Proteomics Bioinf. 13, 278–289. doi: 10.1016/j.gpb.2015.08.002 PMC4678779 26542840

[B41] Samaniego Castruita J. A. Zepeda Mendoza M. L. Barnett R. Wales N. Gilbert M. T. (2015). Odintifier–A computational method for identifying insertions of organellar origin from modern and ancient high-throughput sequencing data based on haplotype phasing. BMC Bioinf. 16, 232. doi: 10.1186/s12859-015-0682-1 PMC4517485 26216337

[B42] Sandhya S. Srivastava H. Kaila T. Tyagi A. Gaikwad K. (2020). Methods and tools for plant organelle genome sequencing, assembly, and downstream analysis. Methods Mol. Biol. 2107, 49–98. doi: 10.1007/978-1-0716-0235-5_4 31893443

[B43] Sanita Lima M. Woods L. C. Cartwright M. W. Smith D. R. (2016). The (in)complete organelle genome: exploring the use and nonuse of available technologies for characterizing mitochondrial and plastid chromosomes. Mol. Ecol. Resour. 16, 1279–1286. doi: 10.1111/1755-0998.12585 27482846

[B44] Servant N. Varoquaux N. Lajoie B. R. Viara E. Chen C. J. Vert J. P.. (2015). HiC-Pro: an optimized and flexible pipeline for Hi-C data processing. Genome Biol. 16, 259. doi: 10.1186/s13059-015-0831-x 26619908 PMC4665391

[B45] Shirasawa K. Itai A. Isobe S. (2021). Chromosome-scale genome assembly of Japanese pear (Pyrus pyrifolia) variety 'Nijisseiki'. DNA Res. 28 (2), 1–6. doi: 10.1093/dnares/dsab001 PMC8092371 33638981

[B46] Sikorskaite S. Rajamaki M. L. Baniulis D. Stanys V. Valkonen J. P. (2013). Protocol: Optimised methodology for isolation of nuclei from leaves of species in the Solanaceae and Rosaceae families. Plant Methods 9, 31. doi: 10.1186/1746-4811-9-31 23886449 PMC3728069

[B47] Song W. Ye T. Liu S. Shen D. Du Y. Yang Y.. (2024). Chrom-pro: A user-friendly toolkit for *de-novo* chromosome assembly and genomic analysis. bioRxiv. doi: 10.1101/2024.03.02.583079

[B48] Tillich M. Lehwark P. Pellizzer T. Ulbricht-Jones E. S. Fischer A. Bock R.. (2017). GeSeq - versatile and accurate annotation of organelle genomes. Nucleic Acids Res. 45, W6–W11. doi: 10.1093/nar/gkx391 28486635 PMC5570176

[B49] Timmis J. N. Ayliffe M. A. Huang C. Y. Martin W. (2004). Endosymbiotic gene transfer: organelle genomes forge eukaryotic chromosomes. Nat. Rev. Genet. 5, 123–135. doi: 10.1038/nrg1271 14735123

[B50] Vaser R. Sovic I. Nagarajan N. Sikic M. (2017). Fast and accurate *de novo* genome assembly from long uncorrected reads. Genome Res. 27, 737–746. doi: 10.1101/gr.214270.116 28100585 PMC5411768

[B51] Vurture G. W. Sedlazeck F. J. Nattestad M. Underwood C. J. Fang H. Gurtowski J.. (2017). GenomeScope: fast reference-free genome profiling from short reads. Bioinformatics 33, 2202–2204. doi: 10.1093/bioinformatics/btx153 28369201 PMC5870704

[B52] Walker B. J. Abeel T. Shea T. Priest M. Abouelliel A. Sakthikumar S.. (2014). Pilon: an integrated tool for comprehensive microbial variant detection and genome assembly improvement. PloS One 9, e112963. doi: 10.1371/journal.pone.0112963 25409509 PMC4237348

[B53] Wang B. Yang X. Jia Y. Xu Y. Jia P. Dang N.. (2022). High-quality arabidopsis thaliana genome assembly with nanopore and HiFi long reads. Genomics Proteomics Bioinf. 20, 4–13. doi: 10.1016/j.gpb.2021.08.003 PMC9510872 34487862

[B54] Wang J. Kan S. Liao X. Zhou J. Tembrock L. R. Daniell H.. (2024). Plant organellar genomes: much done, much more to do. Trends Plant Sci. 29 (7), 754–769. doi: 10.1016/j.tplants.2023.12.014 38220520

[B55] Wang P. Luo Y. Huang J. Gao S. Zhu G. Dang Z.. (2020). The genome evolution and domestication of tropical fruit mango. Genome Biol. 21, 60. doi: 10.1186/s13059-020-01959-8 32143734 PMC7059373

[B56] Wang X. Cheng F. Rohlsen D. Bi C. Wang C. Xu Y.. (2018). Organellar genome assembly methods and comparative analysis of horticultural plants. Hortic. Res. 5, 3. doi: 10.1038/s41438-017-0002-1 29423233 PMC5798811

[B57] Wei W. Schon K. R. Elgar G. Orioli A. Tanguy M. Giess A.. (2022). Nuclear-embedded mitochondrial DNA sequences in 66,083 human genomes. Nature 611, 105–114. doi: 10.1038/s41586-022-05288-7 36198798 PMC9630118

[B58] Xu M. Guo L. Gu S. Wang O. Zhang R. Peters B. A.. (2020). TGS-GapCloser: A fast and accurate gap closer for large genomes with low coverage of error-prone long reads. Gigascience 9 (9), 1–11. doi: 10.1093/gigascience/giaa094 PMC7476103 32893860

[B59] Yu N. Li J. Bao H. Zhang Y. Yang Z. Li F.. (2024). Chromosome-level genome of spider Pardosa pseudoannulata and cuticle protein genes in environmental stresses. Sci. Data 11, 121. doi: 10.1038/s41597-024-02966-1 38267470 PMC10810088

[B60] Zhang W. Yang Y. Hua S. Ruan Q. Li D. Wang L.. (2024). Chromosome-level genome assembly and annotation of the yellow grouper, Epinephelus awoara. Sci. Data 11, 151. doi: 10.1038/s41597-024-02989-8 38296995 PMC10830450

[B61] Zhang X. Zhang S. Zhao Q. Ming R. Tang H. (2019). Assembly of allele-aware, chromosomal-scale autopolyploid genomes based on Hi-C data. Nat. Plants 5, 833–845. doi: 10.1038/s41477-019-0487-8 31383970

[B62] Zhong X. (2020). Assembly, annotation and analysis of chloroplast genomes. Perth, Western Australia, Australia: The University of Western Australia. Available online at: https://research-repository.uwa.edu.au/en/publications/assembly-annotation-and-analysis-of-chloroplast-genomes.

[B63] Zhou W. Armijos C. E. Lee C. Lu R. Wang J. Ruhlman T. A.. (2023a). Plastid genome assembly using long-read data. Mol. Ecol. Resour. 23, 1442–1457. doi: 10.1111/1755-0998.13787 36939021 PMC10354735

[B64] Zhou X. Peng T. Zeng Y. Cai Y. Zuo Q. Zhang L.. (2023b). Chromosome-level genome assembly of Niphotrichum japonicum provides new insights into heat stress responses in mosses. Front. Plant Sci. 14. doi: 10.3389/fpls.2023.1271357 PMC10619864 37920716

[B65] Zhu F. Yin Z. T. Wang Z. Smith J. Zhang F. Martin F.. (2021). Three chromosome-level duck genome assemblies provide insights into genomic variation during domestication. Nat. Commun. 12, 5932. doi: 10.1038/s41467-021-26272-1 34635656 PMC8505442

