# Supplementary figures and images for: Chlomito: a novel tool for precise elimination of organelle genome contamination from nuclear genome assembly
Source: Front Plant Sci. 2024 Aug 27;15:1430443. doi: 10.3389/fpls.2024.1430443 (PMC11385003; doi:10.3389/fpls.2024.1430443)

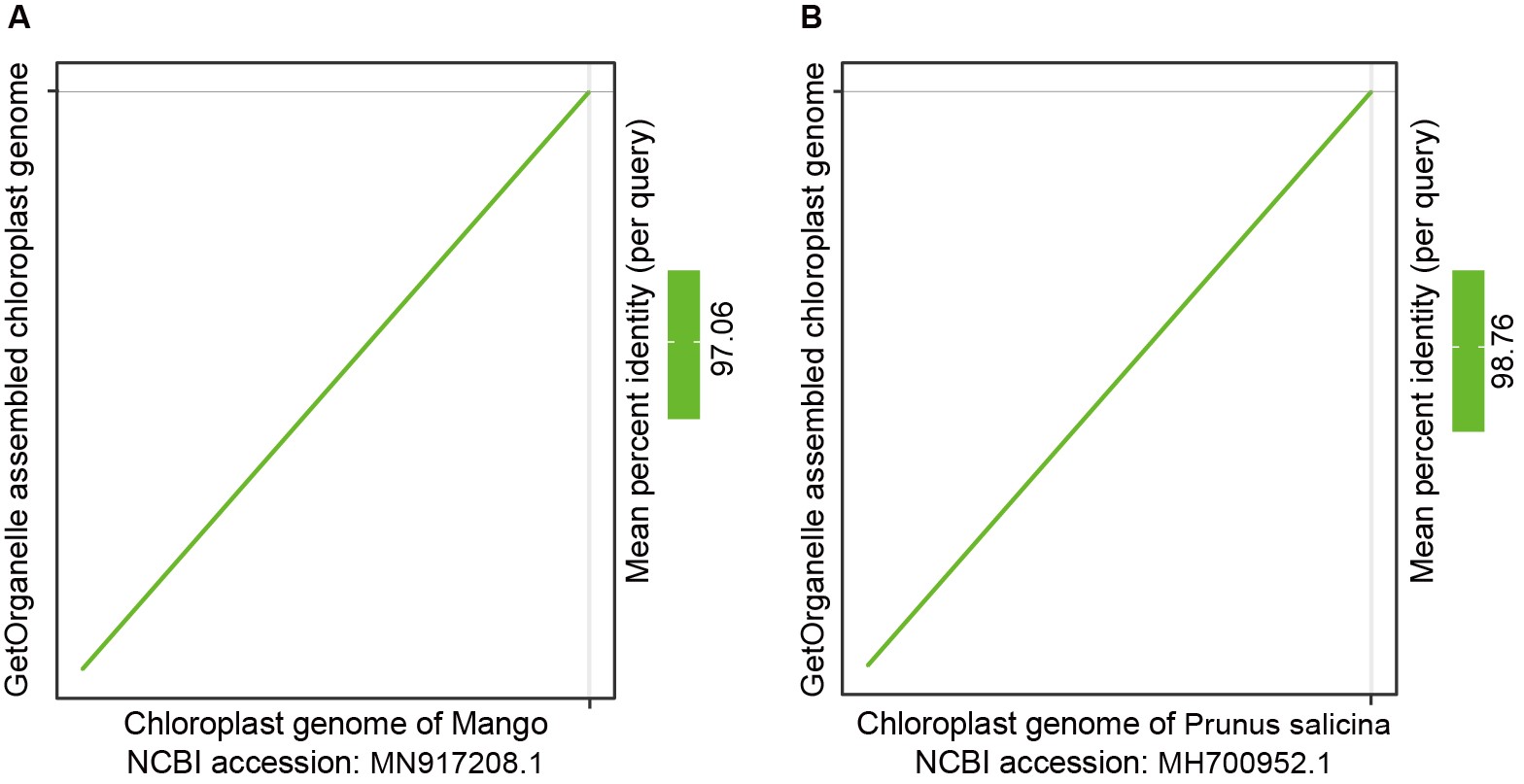

Supplement: Supplementary Figure 1 — High collinearity between GetOrganelle-assembled chloroplast genomes and NCBI reference genomes. The results from Mango (A) and Plum (B) are shown. [file Image1.jpeg]

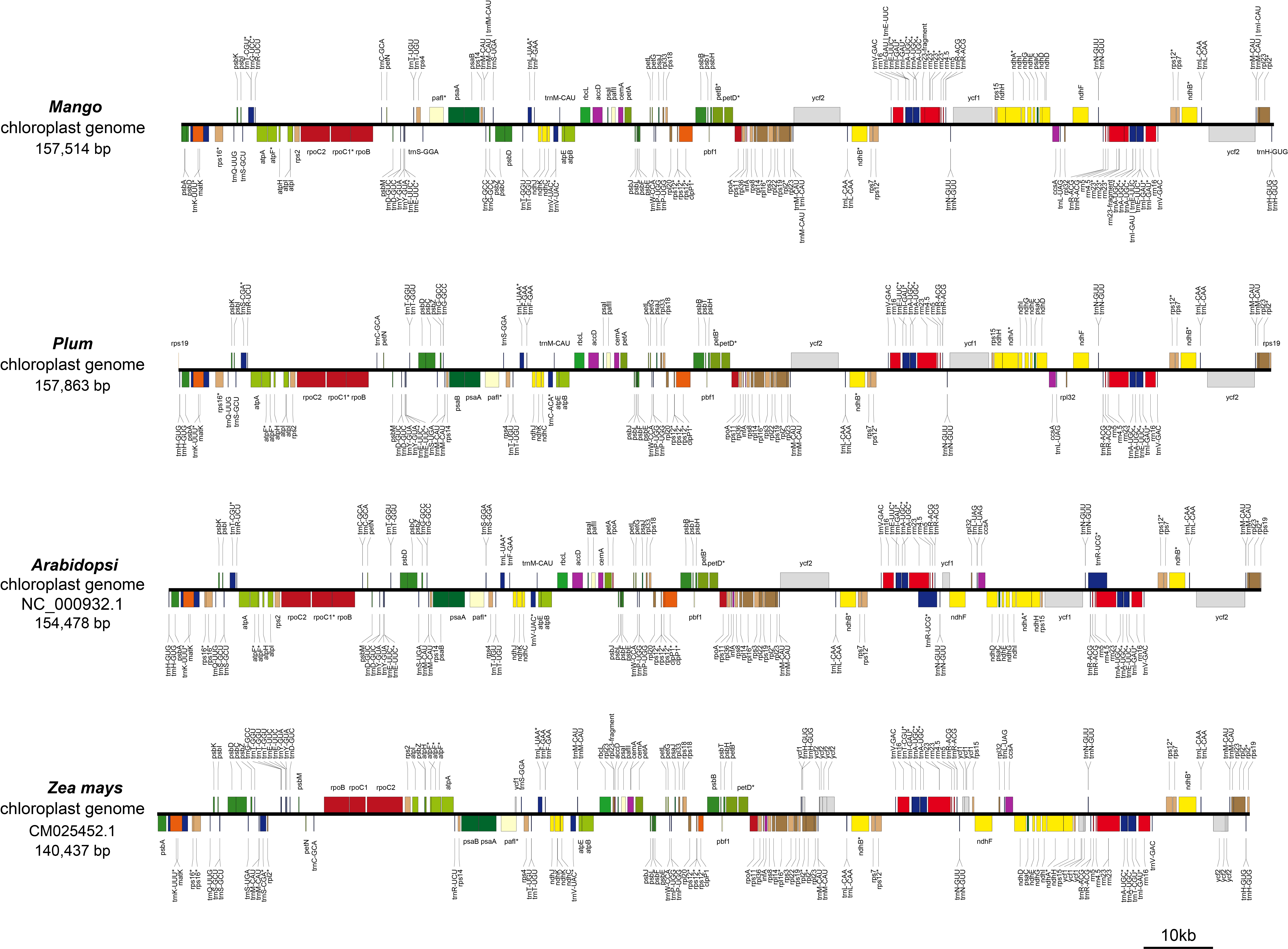

Supplement: Supplementary Figure 2 — Annotation and comparison of chloroplast genomes of Mango, Plum, Arabidopsis, and Zea mays. The chloroplast genomes of these species exhibit high conservation in gene contents and order. [file Image2.jpeg]

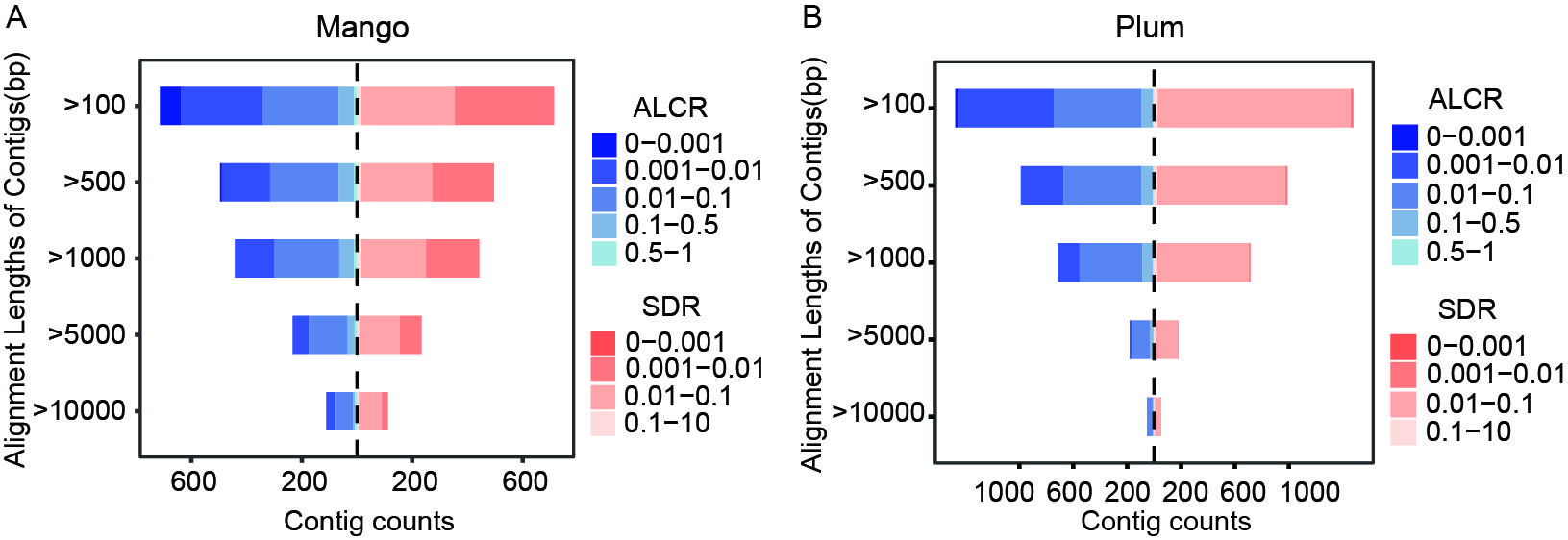

Supplement: Supplementary Figure 3 — Analysis of contigs with chloroplast reference genome alignments in Mango (A) and Plum (B) samples. The alignment length coverage ratio (ALCR, blue bars), sequencing depth ratio (SDR, orange bars), and contig alignment lengths with chloroplast reference genomes are shown for contigs with varying alignment lengths. [file Image3.jpeg]

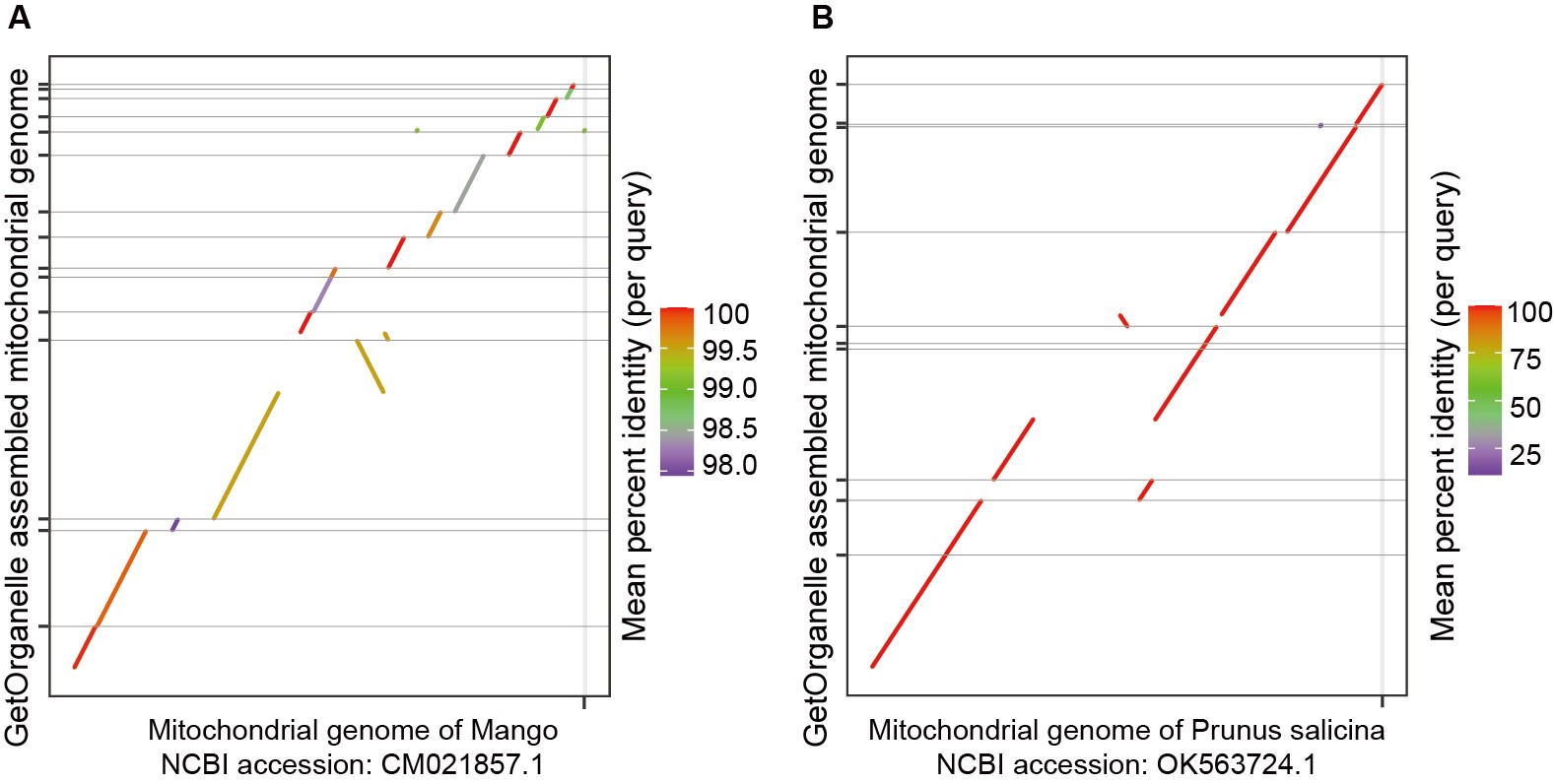

Supplement: Supplementary Figure 4 — Collinearity comparison of mitochondrial genomes of Mango (A) and Plum (B) assembled by GetOrganelle with mitochondrial reference genomes from NCBI. [file Image4.jpeg]

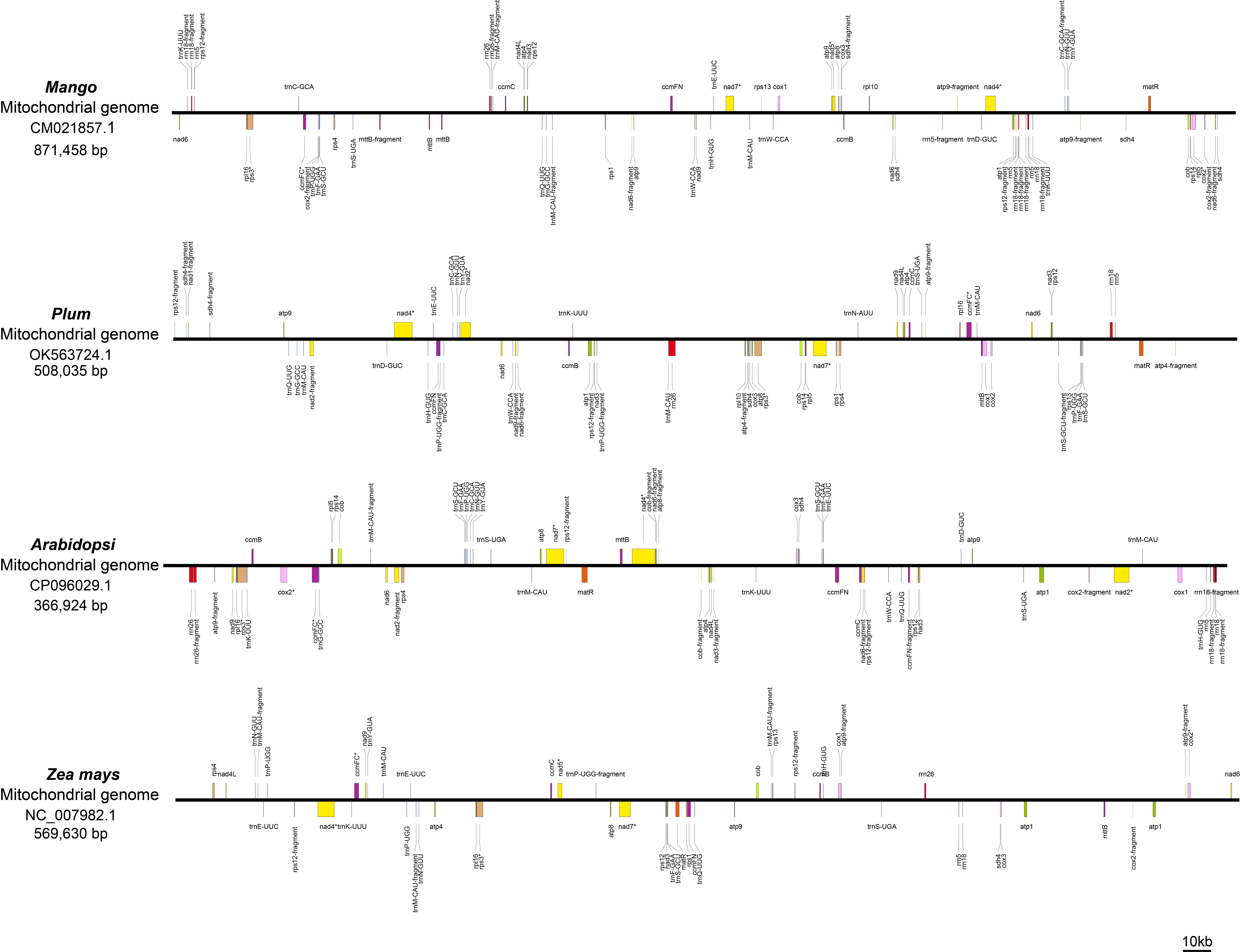

Supplement: Supplementary Figure 5 — Annotation and comparison of mitochondrial genomes of Mango, Plum, Arabidopsis, and Zea mays. In contrast to the conserved chloroplast genomes ( Supplementary Figure S2 ), the mitochondrial genomes of these species show significant variations in gene content and gene order. [file Image5.jpeg]

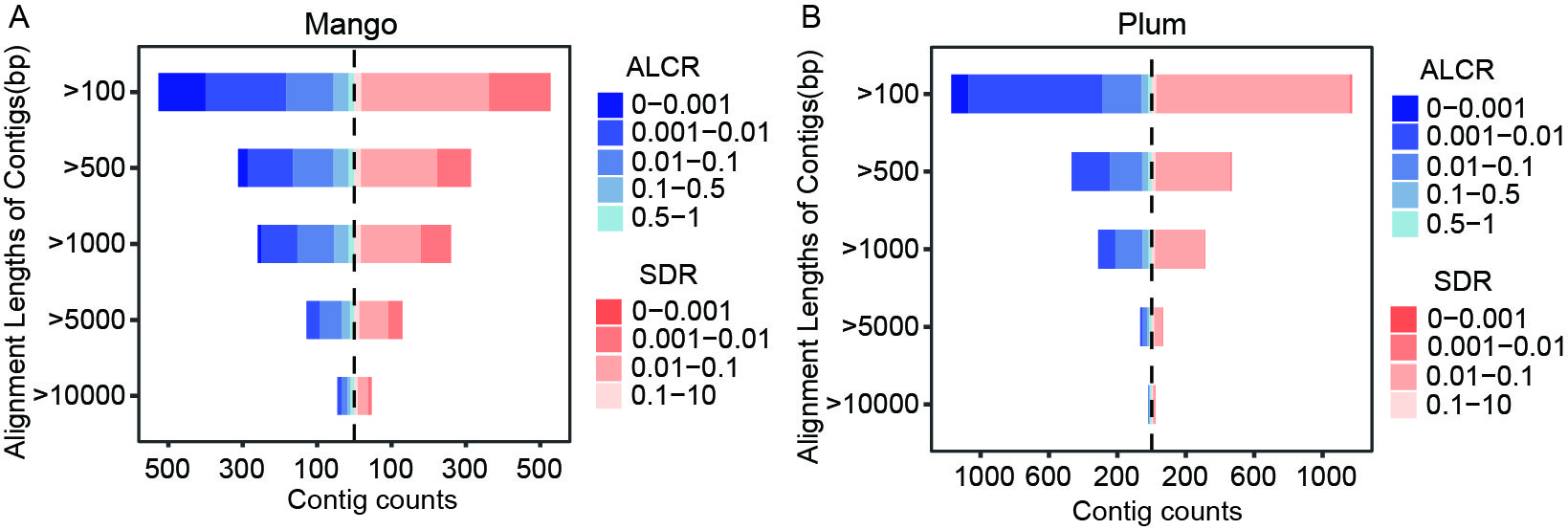

Supplement: Supplementary Figure 6 — Analysis of contigs with mitochondrial reference genome alignments in Mango (A) and Plum (B) samples. The alignment length coverage ratio (ALCR, blue bars), sequencing depth ratio (SDR, orange bars), and contig alignment lengths with mitochondrial reference genomes are shown for contigs with varying alignment lengths. [file Image6.jpeg]

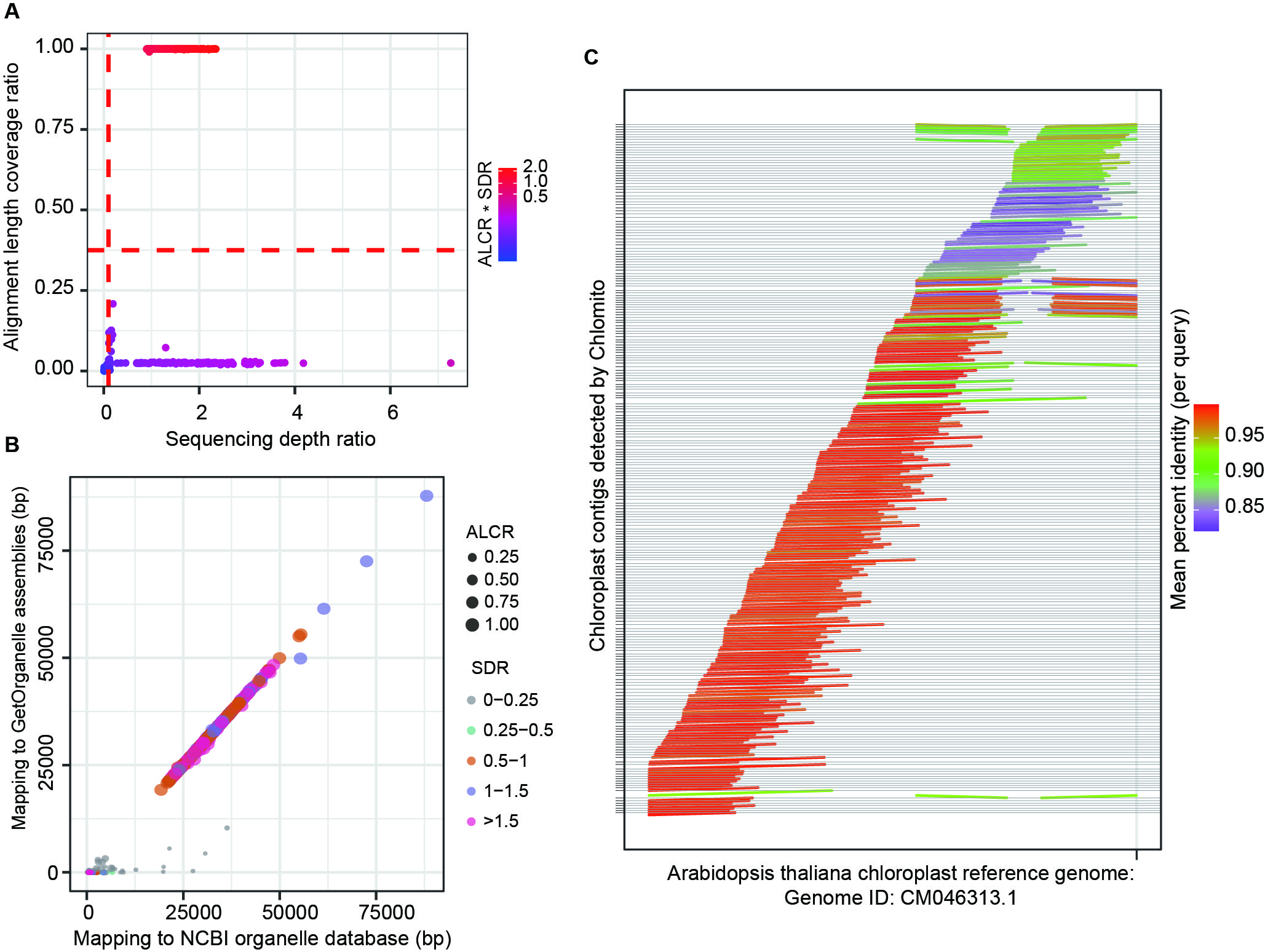

Supplement: Supplementary Figure 7 — Chlomito accurately identifies chloroplast-derived contigs and validates their collinearity with chloroplast reference genome from Arabidopsis. (A) Identification of chloroplast-derived contigs in Arabidopsis assembly based on ALCR and SDR metrics. (B) Alignment lengths of Arabidopsis contigs with chloroplast genomes assembled using GetOrganelle and downloaded from NCBI database. (C) Collinearity analysis of Arabidopsis contigs identified by Chlomito against published Arabidopsis chloroplast reference genome. [file Image7.jpeg]
