# Supplementary material for: Chlomito: a novel tool for precise elimination of organelle genome contamination from nuclear genome assembly
Source: Front Plant Sci. 2024 Aug 27;15:1430443. doi: 10.3389/fpls.2024.1430443 (PMC11385003; doi:10.3389/fpls.2024.1430443)
Supplement: Supplementary Table 1 — Repetitive sequences identified using RepeatMasker in the Plum contigs with low ALCR and high SDR. [file Table1.docx]

**Table S1.** Repetitive sequences identified using RepeatMasker in the Plum contigs with low ALCR and high SDR.

|  | Total length | GC level | Repetitive sequence length |
| --- | --- | --- | --- |
| contig_741 | 3193 bp | 55.28% | 2273 bp (71.19 %) |
| contig_2322 | 8911 bp | 43.87% | 6280 bp (70.47 %) |
| contig_829 | 9400 bp | 47.70% | 6477 bp (68.90 %) |
| contig_710 | 22313 bp | 43.08% | 14894 bp (66.75 %) |
| contig_2344 | 14975 bp | 43.45% | 8704 bp (58.12 %) |
| contig_1293 | 103789 bp | 41.27% | 42073 bp (40.54 %) |
| contig_1115 | 107136 bp | 41.36% | 38336 bp (35.78 %) |
